# Purification of Lepidilines A, B, C, and D from *Lepidium meyenii* Walpers by Centrifugal Partition Chromatography Followed by Semi-Preparative HPLC and Preliminary Evaluation of Anticancer Activity Against Neuroblastoma Cell Lines

**DOI:** 10.3390/molecules30224360

**Published:** 2025-11-11

**Authors:** Dominik Tarabasz, Estera Okoń, Anna Wawruszak, Stavros Beteinakis, Apostolis Angelis, Henry O. Meissner, Leandros A. Skaltsounis, Wirginia Kukula-Koch

**Affiliations:** 1 Department of Pharmacognosy with Medicinal Plants Garden, Medical University of Lublin, 1 Chodzki Str., 20-093 Lublin, Poland; 2 Department of Biochemistry and Molecular Biology, Medical University of Lublin, 1 Chodzki Str., 20-093 Lublin, Poland; estera.okon@umlub.pl (E.O.); anna.wawruszak@umlub.pl (A.W.); 3 Division of Pharmacognosy and Natural Products Chemistry, Department of Pharmacy, National and Kapodistrian University of Athens, Panepistimioupoli Zografou, 15771 Athens, Greece; sbeteinakis@pharm.uoa.gr (S.B.); aangjel@pharm.uoa.gr (A.A.); skaltsounis@pharm.uoa.gr (L.A.S.); 4 NICM Health Research Institute, Western Sydney University, 158-160 Hawkesbury Road, Sydney, NSW 2145, Australia

**Keywords:** *Lepidium meyenii*, *Lepidium peruvianum*, lepidilines, isolation, counter-current chromatography, neuroblastoma, SK-N-AS, extraction, brassicaceae

## Abstract

*Lepidium meyenii* Walpers (syn. *Lepidium peruvianum* Chacon) has been cultivated for centuries in the Peruvian Andes as both a vegetable and a traditional medicine resource. Maca is classified as a superfood and is widely used as a dietary supplement, particularly noted for its potential to enhance endurance, fertility, and endocrine balance. In recent years, there has been a growing interest in the cytotoxic effects of lepidilines and their derivatives; however, these compounds have been less extensively studied due to challenges associated with their isolation. This study aims to establish optimal extraction conditions to enrich lepidiline content in the extracts and to propose an efficient isolation method for four lepidilines using a green purification technique known as Centrifugal Partition Chromatography (CPC). The isolated compounds will be evaluated for their anticancer potential utilizing the MTT assay on SK-N-SH (ATCC^®^ HTB-11™) and SK-N-AS (ATCC^®^ CRL-2137™) neuroblastoma cell lines. The findings indicate that Soxhlet extraction with dichloromethane resulted in the highest recovery of lepidilines, with a content of 10.24% expressed as lepidiline A. The optimal biphasic solvent mixture suitable for CPC chromatographic applications was identified as a combination of chloroform, methanol, and water (4:3:2 *v*/*v*/*v*) containing 60 mM HCl. When utilized in conjunction with semi-preparative high-performance liquid chromatography (HPLC), this method successfully isolated lepidilines A–D, achieving a purity exceeding 95%. Notably, lepidiline B exhibited the highest cytotoxic potential, with an IC50 value of 14.85 µg/mL in SK-N-AS cells.

## 1. Introduction

*Lepidium meyenii* Walpers (Brassicaceae), commonly referred to as maca, is a plant indigenous to the elevated regions of the Peruvian Andes, where it has been utilized by local populations for over two millennia as both a food source and a medicinal agent, enhancing energy and endurance. Due to its adaptogenic properties, it has also been colloquially designated as Peruvian ginseng [1]. The growing interest in maca has been further amplified by the scientific community’s efforts to elucidate its potential to enhance male fertility and its impact on the hormonal system [2,3]. As maca’s popularity continues to rise within the dietary supplement industry, it has become the subject of extensive research focused on its multifaceted bioactivities, resulting in a wealth of scientific literature that expands the understanding of the medicinal properties of its hypocotyls. Current in vitro studies confirm the biological activity of maca extracts, highlighting their neuroprotective, anti-inflammatory, analgesic, immunoregulatory, antitumor, antioxidant, fertility-enhancing, anti-fatigue, gastrointestinal, cardioprotective, antihypertensive, photoprotective, anabolic, and hepatoprotective effects [4].

*Lepidium meyenii* is recognized as a rich source of diverse metabolites characteristic of this plant, which have been demonstrated to induce the aforementioned biological effects. Prominent among these are glucosinolates, alkaloids—including benzylalkylamides and imidazole alkaloids—polyunsaturated fatty acids, and their derivatives, such as macamides, macaenes, and benzamides [5].

**Figure 1:**
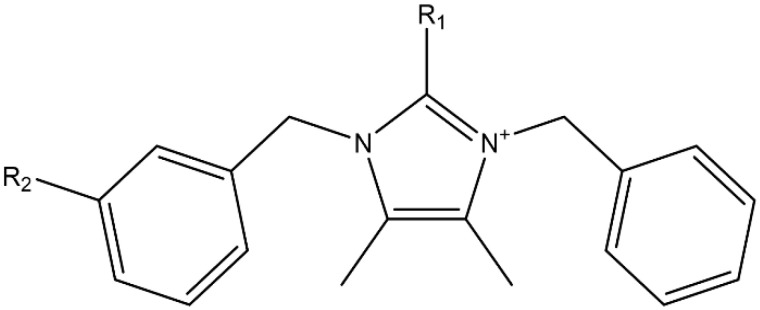
Parent structure of lepidilines A, B, C, and D. Lepidiline A: R_1_: -H; R_2_: -H, Lepidiline B: R_1_: -CH_3_; R_2_: -H, Lepidiline C: R_1_: -H; R_2_: -OCH_3_, Lepidiline D: R_1_: -CH_3_; R_2_: -OCH_3_.

Lepidilines, a class of imidazole alkaloids unique to *Lepidium meyenii*, have received relatively limited attention thus far. Current literature indicates that maca extracts contain lepidilines A, B, C, and D, while Le et al. [6] also identify lepidilines E, F, and G (see Figure 1). However, information regarding the bioactivity of these compounds remains scarce. Cheng et al. [7] demonstrated that lepidiline A enhances endogenous sex hormone levels and promotes fertility by modulating the enzyme 17β-hydroxysteroid dehydrogenase 1 (HSD17B1). In another investigation, lepidiline B exhibited cytotoxic activity against the HL-60 cancer cell line and moderate anti-inflammatory effects in LPS-treated RAW 264.7 macrophages [8] with low cytotoxicity (IC50 > 100 μM) against normal HUVEC cells.

Given the anticancer potential of lepidilines, some studies have suggested leveraging their structural framework as a core for the synthesis of novel, more potent anticancer agents in complexes, such as those with N-heterocyclic carbene gold [9,10], N-methylated derivatives, or fluorine, fluoroalkyl, and fluoroalkoxyl substituted analogs [11], some of which were proven to be potent anticancer drug candidates in the in vitro tests on HL-60 and MCF-7 cells.

The pharmacological potential of lepidilines needs further investigation, necessitating more comprehensive studies to elucidate their capabilities. In light of the previously established anticancer properties of lepidilines, this manuscript aims to present novel data on the effects of these alkaloids on cell lines associated with central nervous system tumors. Such information could augment the already identified memory-enhancing properties attributed to maca in earlier studies [12,13]. However, the recovery and isolation of lepidilines from plant matrices pose significant challenges, particularly due to the tendency of alkaloids to adsorb onto solid adsorbents during chromatographic processes [14]. This phenomenon likely accounts for the limited commercial availability of lepidiline standards and the relatively sparse scientific literature concerning their potential applications in natural product-based therapeutic strategies.

The objective of the current study is to focus on the recovery of lepidilines from the plant matrix by investigating the application of various extraction techniques on crude plant material, aimed at maximizing the yield of lepidilines. For this purpose, several extraction techniques, including ultrasonic bath extraction, supercritical carbon dioxide extraction, Soxhlet extraction, and accelerated solvent extraction (ASE), will be used under varying temperature, cycle duration, and pressure conditions to select the most advantageous technique targeting lepidilines’ recovery from *Lepidium meyenii* tubers. The characterization of the resulting extracts will be conducted using high-performance liquid chromatography coupled with mass spectrometry (HPLC-MS) and diode array detector (HPLC-DAD) methodologies to provide precise data.

Furthermore, the study seeks to propose efficient and scalable protocols for their isolation from the total extract, which will enable high-throughput separation and enhance the availability of these compounds for biological evaluation.

Previous attempts at isolating lepidilines were based on the application of conventional, costly, and resource-consuming chromatographic techniques that included a multi-step separation on macroporous resin, Al_2_O_3_, and C-18 columns and were succeeded by preparative HPLC purification [15].

In the course of this study, we propose to utilize the CPC-HPLC preparative approach that is anticipated to yield sufficient quantities of these metabolites for subsequent biological studies while minimizing solvent consumption and maximizing the selectivity of chromatographic conditions. Centrifugal partition chromatography (CPC) represents a noteworthy green separation technique distinguished by its lack of a solid stationary phase, and because of this fact, it minimizes the risk of compound loss during separation. The diverse conditions for CPC-based separation processes and the multitude of biphasic solvent systems applicable in this technique ensure high selectivity and efficiency in isolating challenging components, such as alkaloids [16].

In the end, the isolated and purified lepidilines will subsequently be evaluated for their cytotoxic activity against SK-N-SH and SK-N-AS neuroblastoma cancer cell lines, filling the gaps in the biological potential of these underestimated molecules.

## 2. Results

### 2.1. Identification of Lepidilines A–D by HPLC-ESI-QTOF-MS Approach

**Figure 2:**
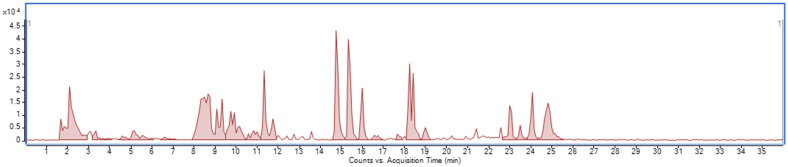
LC/MS BPC (ESI+) MS spectra of the total extract from the red variety of *Lepidium meyenii*.

In this study, the composition of compounds in the extract of *Lepidium meyenii* obtained through ultrasonic bath extraction is presented in Figure 2. For the purpose of this experiment, the LC-MS method was adapted to identify four lepidilines (A–D). The identification of these compounds was based on data from the MS detector and information available in the literature, with particular attention paid to fragmentation data (Table 1) for the main ions detected. Additionally, the retention time and the fragmentation pattern of lepidiline A were compared with those of the corresponding reference standard.

### 2.2. Extraction Optimization of Lepidilines from Maca Tubers

#### 2.2.1. Accelerated Solvent Extraction (ASE) for the Recovery of Imidazole Alkaloids from *Lepidium meyenii*

The elevated pressure and increased temperature conditions provide a more effective penetration of the solvent to the plant matrix, resulting in an increased efficiency of the extraction process [17]. This study elaborated on optimizing the ASE-based extraction protocol to recover lepidilines A, B, C, and D from maca tubers more efficiently.

**Figure 3:**
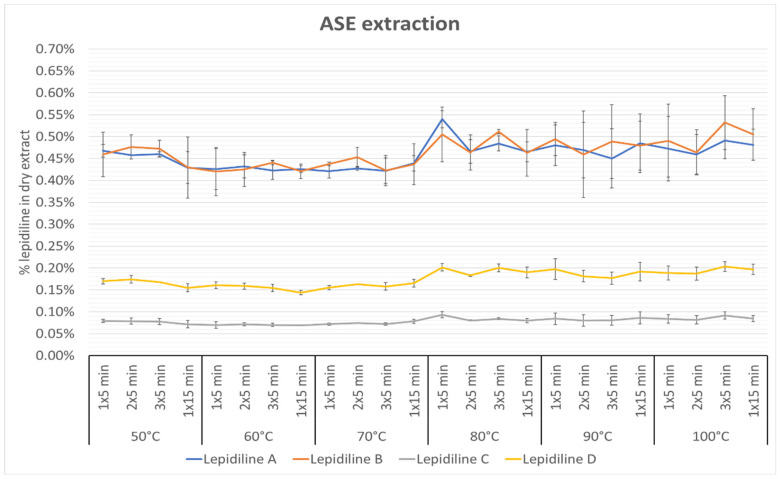
The effect of temperature, cycle numbers, and extraction length on the isolation of lepidilines from *Lepidium meyenii* extract. The data are presented as the mean ± standard deviation (±SD). The percentage value of every compound is expressed as lepidiline A content (*n* = 3). See Table S2 for the exact percentage values of lepidiline and Table S3 for their SD values.

Figure 3 represents the changes in lepidilines’ concentrations in the dry extract obtained using the mentioned technique and show the impact of temperature, number of cycles, and length of extraction on the content of lepidilines in the 50% methanol extract. As presented below, the temperature is a critical factor in the extraction process of imidazole alkaloids from maca. It can, therefore, be assumed that as the ASE extraction temperature increases, the lepidilines concentration also increases. Interestingly, the number of extraction cycles and the cycle length did not significantly affect the extraction of alkaloids. In the obtained results, the most effective extraction conditions for lepidiline A, and C were one 5-min cycle at 80 °C, for lepidiline B—three 5-min cycles at 100 °C, whereas for lepidiline D three 5-min cycles at 100 °C. The obtained extraction yields for these compounds expressed as lepidiline A were: 0.54% (SD = 0.02%), 0.532% (SD = 0.06%), 0.093% (SD = 0.01%), and 0.203% (SD = 0.01%), for lepidiline A, B, C, and D, respectively. Lepidilines were extracted with a similar efficiency. This result may be due to their close chemical structure. The smallest content of these compounds was observed in the extracts prepared at 70 °C in one 5-min cycle for lepidiline A, at 60 °C in one 5-min cycle for lepidilines B and C, whereas in one 15-min cycle at 60 °C in the case of lepidiline D. The best conditions for extracting all lepidilines together were proved at 80 °C in one 5-min cycle and at 100 °C in three 5-min cycles.

#### 2.2.2. Extraction in Soxhlet Apparatus

**Figure 4:**
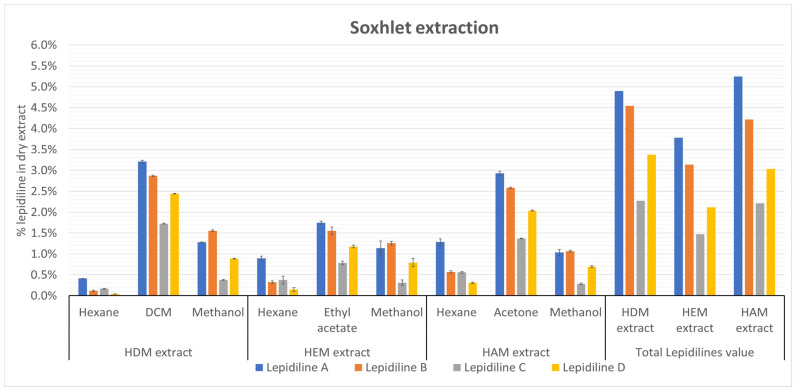
The impact of solvent selection on the extraction of lepidilines in a Soxhlet apparatus. The three visible, separated sections correspond to three extraction processes using fresh batches of pulverized maca. Each extract consists of three solvents, the first (hexane) and the third (methanol) being typical. Finally, the total lepidilines extraction value was summarized for each extract. The data are presented as the mean ± standard deviation ±SD). All quantitative results are expressed as lepidiline A concentration (*n* = 3). See Table S4 for the exact percentage values of lepidiline and Table S5 for their SD values.

In the case of Soxhlet extraction the impact of differently polar solvents was tested. As presented in the Figure 4 the most advantageous solvents for targeted extraction of lepidilines were: DCM > acetone > ethyl acetate in the descending order. The first was able to extract 10.24% of lepidilines from the plant matrix. For the rest, 8.91% and 5.26% of lepidilines were extracted, respectively. The extraction profile of lepidilines for the three solvents mentioned above is identical in the following order: lepidiline A > B > D > C. The apolar solvent—hexane—extracted the lowest values of lepidilines compared to other used solvents, obtaining from 0.74% to 2.72% of alkaloids. The extraction profile of lepidilines with hexane was as follows: lepidiline A > C > B > D. On the other hand, the most polar solvent, methanol, had a different extraction profile of lepidilines, namely lepidiline B > A > D > C. The best solvent combinations to extract lepidilines were those with dichloromethane and acetone.

#### 2.2.3. Supercritical Fluid Extraction of *L. meyenii* Hypocotyls (CO_2_ Extraction)

**Figure 5:**
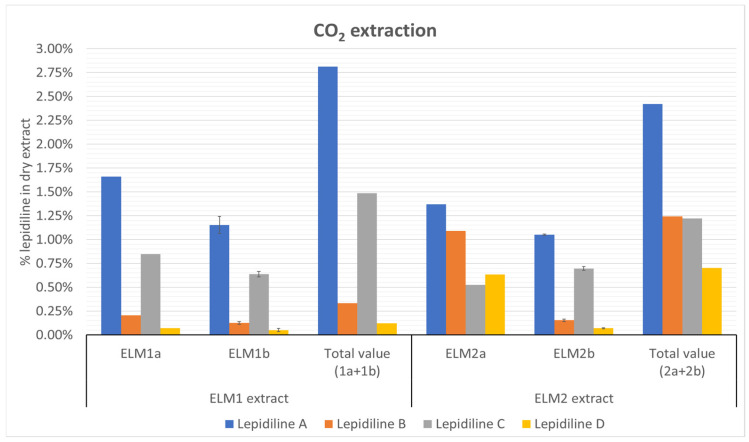
The influence of different experimental conditions on the supercritical CO_2_ extraction of lepidilines. ELM1a—SFE extract with CO_2_ at 150 bar; ELM1b—SFE extract obtained with 5% IP and 95% CO_2_ (*n* = 3); ELM2a—SFE extract with CO_2_ at 250 bar; ELM2b—SFE extract obtained with 5% IP and 95% CO_2_ (*n* = 3).

The experiment showed that increasing the polarity with isopropanol at the tested pressures did not significantly affect the extraction of lepidilines. More importantly, it demonstrated that isopropanol extracts contained a smaller quantity of lepidilines, proving the capability of CO_2_ itself to recover these compounds from the plant matrix. The addition of isopropanol to the extraction conditions was a less efficient process for the extraction of lepidilines than initially assumed, and compared to the extraction attempt without isopropanol. One thing worth noting is that at the second extraction attempt at a higher pressure and without of 5% IP, an increased extraction of lepidilines B and D was observed (see Figure 5). This may be due to the polarity of these molecules. Namely, these two lepidilines have a methyl group in position 2 of the imidazole ring, which gives them an additional apolar character. The ELM1 extract yielded a total of 2.75% lepidiline A, 0.33% lepidiline B, 1.48% lepidiline C, and 0.12% lepidiline D. In contrast, the ELM2 extract contained less lepidiline A (2.42%) and lepidiline C (1.22%) compared to ELM1. Still, it had higher levels of lepidiline B (1.24%) and lepidiline D (0.7%). All percentages are expressed as lepidiline A equivalents (see Figure 5 and Table S6 in the Supplementary File and Table S7 for their SD values).

In the end, a direct comparison of all extraction techniques’ efficiency was performed to show the most favorable ones related to the recovery of lepidilines from plant material. Statistical significance analysis revealed that Soxhlet Extraction and Supercritical CO_2_ Extraction (SFE) were markedly more effective in isolating lepidilines than Accelerated Solvent Extraction (ASE). As it can be seen in the Tables S7 and S8 in the Supplementary File (and explained by the following Figures S1–S6), the columns corresponding to Soxhlet and SFE showed a higher number of significance levels (***), clearly indicating superior extraction performance. The use of Soxhlet and SFE enabled a more efficient recovery of lepidilines A–D from the plant material, which may be attributed to the prolonged solvent contact time in the Soxhlet method and the high solvation capacity of supercritical CO_2_ for lipophilic compounds. Consequently, both Soxhlet Extraction and SFE can be considered more effective techniques for the isolation of lepidilines than ASE. However, among ASE extracts, the process conducted for 5 min at 80 °C was selected as a more advantageous one at * *p*  <  0.05, ** *p*  <  0.01 levels.

### 2.3. Purification of Lepidilines from L. peruvianum Chacon Tuber Extracts by CPC Chromatography

#### 2.3.1. Selection of the Biphasic System

Centrifugal Partition Chromatography was selected as a green, selective, high-throughput technique for isolating lepidilines from maca hypocotyls on a preparative scale. The first step in elaborating a new separation method is selecting a proper mixture of solvents used to construct the biphasic solvent mixture that will selectively influence each of the target molecules. For the extract’s fractionation, various ratios of chloroform, methanol, and water-containing systems were tested as shown in Table 2.

Additionally, the concentration of a modifier, namely: hydrochloric acid (HCl) has been optimized on a single solvent system, to best suit the separation of lepidilines and reduce the compounds’ tailing throughout the column. The tests revealed that adding HCl to the biphasic system enhanced the affinity of lepidilines towards the lower phase, resulting in a more significant difference in the partition coefficients (K_D_) between the analogs. As a result, higher concentrations of HCl, precisely at 60 mM, markedly increased the system selectivity and resulted in the optimal K_D_ values for the lepidilines. After all, the biphasic solvent system composed of chloroform: methanol: water (4:3:2 *v*/*v*/*v*) with 60 mM HCl was selected for the separation on the chromatograph.

#### 2.3.2. Isolation of Lepidilines A, B, C, and D by CPC Chromatography

**Figure 6:**
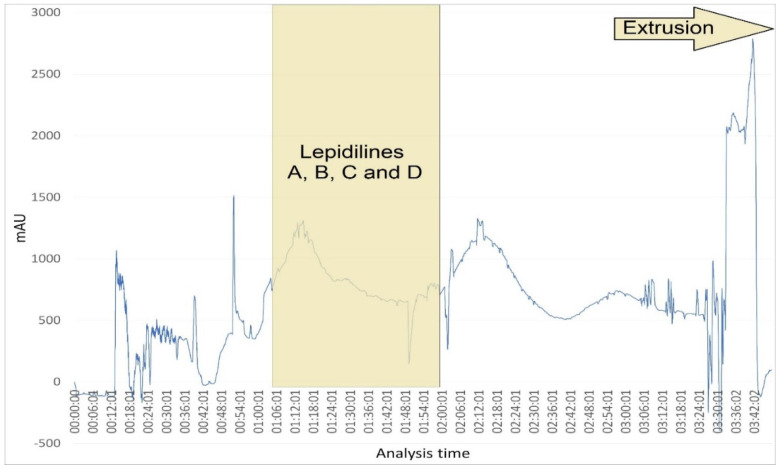
CPC chromatogram of the fraction rich in lepidilines observed at 230 nm. Solvent system: CHCl_3_/MeOH/H_2_O (4/3/2, *v*/*v*/*v*) with 60 mM HCl. Separation conditions: flow rate 4 mL/min and rotation speed 1200 RPM. Extrusion with a stationary phase after 180 min.

Within 220 min, 3 g of lepidilines-rich fraction was separated in the ascending mode using a previously selected biphasic solvent system (see Figure 6). In this elution mode, the compounds eluted from the column with an increasing polarity, that is, starting from lepidiline A, through lepidilines B and C, to lepidiline D. To ensure the best retention of the stationary phase, the flow rate of the mobile phase was set to 4 mL/min and the column rotation speed to 1200 RPM. The peak at about 50 min corresponded to lepidiline E. Lepidilines A–D eluted from the column after 65 min and up to 120 min. The 122 fractions, volumed 5 mL, were concentrated at a temperature of 45 °C to dryness and subjected to HPLC-MS analysis. The purity of lepidilines-containing fractions was later increased in the subsequent purification step that employed the semi-preparative HPLC purification.

#### 2.3.3. Purification of Lepidilines A, B, C, and D by Semi-Preparative High-Performance Liquid Chromatography and Qualitative LC/MS Analyses of *Lepidium meyenii* Extract and Isolated Lepidilines A–D

**Figure 7:**
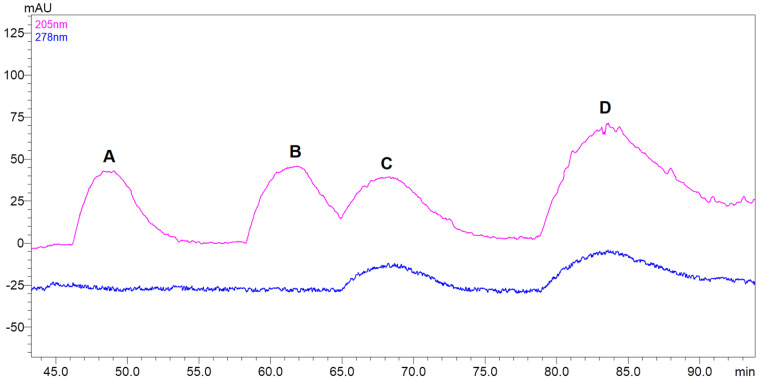
A sample chromatogram of lepidilines A, B, C, and D from the semi-preparative HPLC-based purification. The chromatogram’s letters A, B, C, and D correspond to lepidilines A, B, C, and D, respectively. The detection wavelength, 205 nm (pink) and 278 nm (blue); flow rate, 20 mL/min; mobile phase A: water with 5 mM ammonium acetate, B: methanol: acetonitrile 8:2 (*v*/*v*), were both acidified to a concentration of 0.2% (*v*/*v*) with formic acid.

The applied chromatographic conditions provided a proper chemical environment for the separation of the compounds of interest. The fifteen-milliliter-volumed fractions of lepidilines A–D were collected at the preparative HPLC chromatograph at 205 and 278 nm wavelengths. Lepidiline A was eluted from the column between 46 and 54 min (Figure 7), whereas lepidiline B was eluted from 58 to 65 min. 

**Figure 8:**
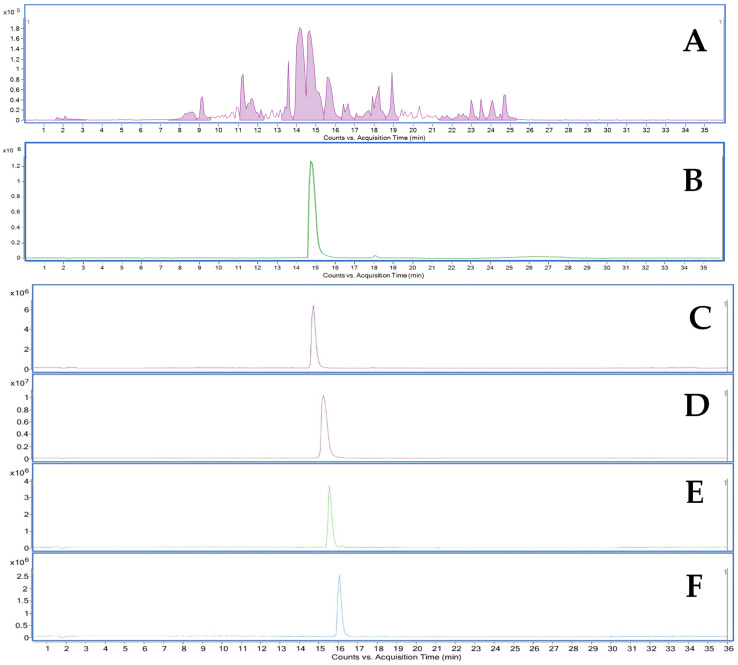
LC/MS BPC (ESI+) MS spectra of the lepidilines-rich fraction (**A**) and the reference compound—lepidiline A (**B**). Purified lepidilines A–D are also shown in the figure and correspond to letters (**C**–**F**), respectively. All lepidilines are >95% pure.

Since lepidiline C was also visible at 278 nm, it was possible to successfully discriminate between lepidiline B and C, which was the biggest challenge in optimizing the separation conditions. As a result, lepidiline C was collected between 66 and 74 min of the run. Lepidilines C and D differ from lepidilines A and B by the presence of a 3-methoxybenzyl group due to which we can probably observe them at an additional wavelength i.e., 278 nm. Finally, lepidiline D was collected between 79 and 92 min. As a result, four compounds—lepidilines A, B, C and D were successfully obtained at high purity exceeding 95% as indicated by the PDA detector peak purity study. The collected yields were 3.96 mg of lepidiline B, 1.93 mg of lepidiline C, and 2.71 mg of lepidiline D. The purified lepidilines were identified by analyzing MS and MS/MS spectra (see Supplementary File Figure S7 for MS/MS spectra of lepidilines A–D) and corresponding fragmentation patterns. Additionally, identification was supported by referencing multiple publications that employed mass spectrometric analyses and NMR (Figure 8 and Table 1) for these compounds. Only lepidiline A was validated by comparison with the MS and MS/MS spectra of a reference compound to confirm its structure. The remaining derivatives were identified based on their similar fragmentation patterns and the high-resolution mass measurements obtained from the recorded analyses.

### 2.4. Bioactivity Assessment in Cells

**Figure 9:**
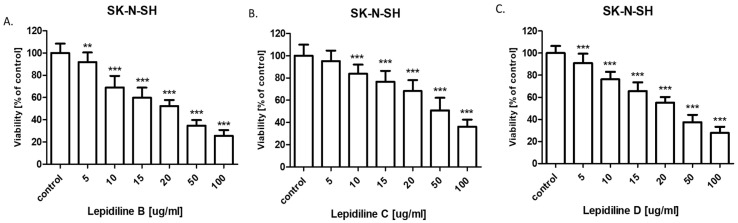
Effect of lepidiline B (**A**), C (**B**), D (**C**) on the viability of SK-N-SH (ATCC^®^ HTB-11™) neuroblastoma cell lines after 96 h with 5–100 μg/mL of the substance in the MTT assay. The data are presented as the mean ± standard deviation (±SD) from 3 independent experiments (*n* = 18); one-way ANOVA, Tukey’s post-hoc testing; ** *p* < 0.01, *** *p* < 0.001.

**Figure 10:**
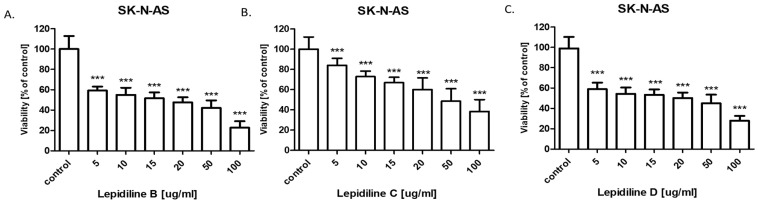
Effect of lepidiline B (**A**), C (**B**), D (**C**) on the viability of SK-N-AS (ATCC^®^ CRL-2137™) human neuroblastoma cell lines after 96 h with 5–100 μg/mL of the substance in the MTT assay. The data are presented as the mean ± standard deviation (±SD) from 3 independent experiments (*n* = 18); one-way ANOVA, Tukey’s post-hoc testing; *** *p* < 0.001.

The cytotoxic activity of lepidiline B, C, and D for the SK-N-SH and SK-N-AS human neuroblastoma cell lines was determined using the MTT assay to calculate the IC_50_ value. The IC_50_ values for the studied neuroblastoma cell lines are presented in Table 3. The SK-N-SH and SK-N-AS cells were exposed to control or increasing (5–100 µg/mL) concentrations of lepidiline B, C, and D. All lepidilines administered individually inhibited cell viability in both analyzed cell lines in a dose-dependent fashion (Figure 9 and Figure 10). The cytotoxic effect of all lepidilines was stronger in SK-N-AS than in SK-N-SH cells. Interestingly, both cell lines were the most sensitive to lepidiline B treatments among all analyzed lepidilines (IC_50_ = 25.75 µg/mL for SK-N-SH and IC_50_ = 14.85 µg/mL for SK-N-AS cells) and the least sensitive to lepidiline C (IC_50_ = 52.07 µg/mL for SK-N-SH and IC_50_ = 44.95 µg/mL for SK-N-AS cells).

## 3. Discussion

The hypocotyls of *Lepidium peruvianum* have gained global recognition for their dual application as both a vegetable and a functional food. Given its widespread consumption, this plant presents a significant source of metabolites with potential applications in the pharmaceutical industry [18]. Currently, the most prominent pharmaceutical applications of *Lepidium peruvianum* center around its phytoestrogenic properties. An increasing number of studies underscore its therapeutic potential, attributable to a diverse array of bioactive compounds, including macamides, glucosinolates, lepidilines, and thiohydantoins. Notably, macamides are particularly interesting due to their influence on hormonal balance and their potential to alleviate symptoms associated with andropause. Conversely, glucosinolates, such as glucotropaeolin, exhibit anti-inflammatory properties and may aid the body in combating cancer [19]. Among the bioactive compounds present, macamides and macaenes are unique to maca and have demonstrated significant neuroprotective and antidepressant effects. These effects are believed to be linked to inhibiting fatty acid amide hydrolase (FAAH), which modulates the endocannabinoid system [20,21]. Furthermore, maca is abundant in glucosinolates, such as glucotropaeolin, precursors to isothiocyanates recognized for their anti-inflammatory and anticancer activities [20]. Particularly noteworthy are the imidazole alkaloids known as lepidilines, which have exhibited cytotoxic effects against various human cancer cell lines in vitro, suggesting their potential utility in future anticancer therapies [20].

The isolation of lepidilines from *Lepidium meyenii* remains a significant challenge for researchers. Optimizing extraction methods for these compounds is critical to obtaining them pure, facilitating their potential use in pharmaceuticals. Maca could emerge as a key botanical resource for addressing hormonal, cancer, and neurodegenerative disorders through such investigations. The primary objective of this research was to establish optimal conditions for the recovery of pharmacologically active nitrogen-containing compounds, specifically lepidilines A, B, C, and D, from the hypocotyls of *Lepidium peruvianum* using scalable extraction techniques. These included accelerated solvent extraction (ASE), supercritical extraction with carbon dioxide and/or isopropanol, and Soxhlet extraction, alongside proposing an efficient protocol for separating these compounds. The isolated lepidilines could ultimately be evaluated for their anticancer potential against neuroblastoma cell lines. The initial phase of the study focused on optimizing the extraction process and elucidating the impact of various parameters on the recovery of these alkaloids. The ASE extraction step, conducted with a single extracting solvent, indicated that temperature exerted the most significant influence on the lepidiline content in the extract, surpassing other variables such as extraction duration and cycle number. It was observed that elevated temperatures enhanced lepidiline extraction, likely due to increased solubility of the alkaloids and improved solvent penetration through plant cell membranes, consistent with previous studies on alkaloid recovery [1]. Higher temperatures correlate with an expanded area of solvent contact with the solid surface, whereas lower temperatures restrict solvent permeability and reduce molecular solubility [22]. In the case of ASE extraction, optimal results were achieved at temperatures between 80–100 °C, where non-thermal degradation of the alkaloids was detected. The lepidilines A and B extraction yields were consistent across all tested temperature ranges, extraction durations, and cycle numbers. At the same time, lepidiline D was observed in higher concentrations, followed by lepidiline C. The study demonstrated that the type of solvent played a pivotal role in the recovery of lepidilines from the plant matrix. The solubility, dissolution, and mass transfer of these components were contingent upon the polarity and nature of the solvent, as previously established in the scientific literature [1]. Although prior studies indicated the presence of lepidilines in water and alcoholic extracts [23], dichloromethane (DCM) emerged as the most effective solvent for lepidilines’ extraction in this study, as confirmed by the Soxhlet extraction protocol. The DCM extracts contained 3.21% ± 0.04%, 2.87% ± 0.01%, 1.72% ± 0.01%, and 2.44% ± 0.01% of lepidines A, B, C, and D, respectively. Additionally, acetone yielded satisfactory results, though with approximately 10% lower concentrations of lepidilines than DCM extraction. This suggests a low to medium polarity of lepidilines and indicates superior dissolution of these compounds in polar organic solvents relative to alcohols.

The supercritical CO_2_ extraction method demonstrated that carbon dioxide could recover the target alkaloids in appreciable quantities, yielding approximately 1% for lepidiline A, 0.1% for lepidiline B, 0.6% for lepidiline C, and 0.05% for lepidiline D. Interestingly, the addition of isopropanol as a co-solvent did not significantly enhance the extraction yield of lepidilines, indicating the higher efficiency of carbon dioxide as a solvent. Moreover, employing a higher extraction pressure of 250 bar proved more effective for extracting lepidilines B and D than 150 bar, possibly due to their apolar characteristics, which align with the expected behavior of low-polarity compounds.

When comparing the extraction techniques employed in this research, the Soxhlet apparatus facilitated the attainment of high concentrations of lepidilines from powdered maca tubers. A notable advantage of this method is its capacity for continuous and repetitive solvent washing of the plant matrix, likely contributing to enhanced extraction efficiency. The boiling points of the solvents utilized in this protocol—68.7 °C for hexane, 39.6 °C for dichloromethane, 77.1 °C for ethyl acetate, 56.05 °C for acetone, and 64.7 °C for methanol—did not exceed 80 °C, thereby preventing the decomposition of the target compounds. Furthermore, considering the stability of lepidilines at elevated temperatures during ASE extraction, which reached 100 °C in this study, it can be inferred that these imidazole alkaloids are chemically stable and do not undergo degradation at these temperatures. This characteristic mitigates a potential drawback of Soxhlet extraction: compound degradation due to prolonged exposure to heat. Importantly, this method is classified as a “green” extraction technique due to low energy and solvent consumption.

The combination of reduced solvent usage, chemical stability of lepidilines at the tested temperatures, and high lepidiline concentrations compared to other extraction techniques employed in this study positions Soxhlet extraction as the most suitable method for lepidiline extraction, with promising potential for successful scaling up. It is noteworthy that the introduction of elevated temperatures or the implementation of enhanced extraction protocols were factors facilitating improved recovery of polyphenols from maca hypocotyls, as evidenced by an antiradical study conducted on L. peruvianum by Dzięcioł et al. [24]. Similar conclusions were drawn from Chen et al. [25], who utilized petroleum ether to extract the least polar constituents of maca, such as macamides and macaenes. The extraction conducted by these authors at temperatures close to the solvent’s boiling point, approximately 40 °C, yielded the highest recovery of these constituents.

The optimization of extraction processes yielded a substantial extract enriched with lepidilines for subsequent purification. The scientific literature presents only a limited number of examples of lepidilines recovery from extracts. Among these, the study by Cui et al. [23] is noteworthy. In their isolation protocol, the authors utilized the chemical characteristics of lepidilines, which can exist in two forms: water-soluble salts and water-insoluble bases. Their purification process involved a series of liquid-liquid extraction steps and precipitation stages to separate lepidilines from other metabolites in the extract. Although successful, this procedure was time-consuming and associated with a significant percentage of sample loss. Another multi-step lepidiline recovery process was reported by Jin et al. [15]. After converting all alkaloids into salt forms, the authors fractionated the extract using microporous resins with acidified eluting solutions. This was followed by additional purification on an aluminum oxide column, semi-preparative HPLC, and a reverse-phase solid-phase extraction method. Previous isolation protocols, while effective, resulted in limited yields of lepidilines due to substantial sample losses. Thus, developing an efficient isolation protocol capable of accommodating high sample loadings and delivering significantly increased quantities of lepidilines for bioactivity studies became essential. Centrifugal Partition Chromatography (CPC) emerged as an appropriate technique for achieving this objective. This method has frequently been employed to purify alkaloids from crude plant extracts. Various review papers have documented prior evidence of alkaloid purification through CPC [26,27]. The technique’s reliance on no solid support facilitates the purification of alkaloids prone to irreversible adsorption onto solid phases. The low sample loss ratio, low operational temperatures, and the versatility of solvents applicable to the separation process contribute to the proposed conditions’ high selectivity and purification efficiency [28].

In this study, the ability to inject up to 3 g of a lepidiline-rich fraction into a 250 mL column was a significant achievement. A biphasic solvent system composed of chloroform, methanol, and water (4:3:2 *v*/*v*/*v*) was employed based on preliminary partition coefficient (KD) screening and existing literature. These solvents were previously found effective for fractionating extracts containing similarly structured alkaloids, including berberine, epiberberine, coptisine, and palmatine [29,30]. Incorporating buffers into the final biphasic solvent composition enhanced the system’s selectivity for lepidilines. Despite their chemical similarities (Figure 1), the system maintained sufficient selectivity, allowing their distinct elution from the chromatographic column. Lepidilines are the most recognizable imidazole alkaloids from maca. Due to the structural similarities among these four compounds, selecting an appropriate solvent system with differentiated KD values conducive to efficient separation proved challenging. The solvent system selection was chosen by preliminary test tube screenings, which indicated that the chloroform/methanol/water mixture (4:3:2 *v*/*v*/*v*) yielded promising resolution of the target compounds. Multiple trials incorporating acids and bases were conducted, with the optimal separation of lepidilines achieved upon adding concentrated HCl to the solvent system. The optimization process involved a comparative evaluation of various biphasic solvent systems (summarized in Supplementary Table S1), most of which failed to provide adequate selectivity among the four primary lepidilines. A critical breakthrough in the analysis was the observation that adding hydrochloric acid enhanced the affinity of lepidilines for the upper phase of the biphasic solvent system. The most pronounced change in KD values was noted for lepidiline D, followed by lepidilines C, B, and A. This trend may be attributed to structural variations among the compounds. Lepidilines D and C possess a 3-methoxybenzyl group, which likely enhances interactions with the polar phase. Furthermore, lepidiline D contains an additional methyl group compared to lepidiline C, potentially affecting charge distribution and spatial arrangement, thus increasing its solubility in the polar phase upon acidification. In contrast, lepidilines A and B exhibit greater symmetry and fewer modifications, resulting in less pronounced changes in their phase distribution. The optimized concentration and presence of acid had a substantial regulatory impact on the KD values of lepidilines. Based on these findings, CPC chromatography is established to effectively purify lepidilines from plant matrices, offering scalability, low solvent consumption, and minimal sample loss.

Semi-preparative HPLC was subsequently employed to obtain pure lepidilines from the CPC fractions. Initially, an acetonitrile-water system was utilized with a 13 mL/min flow rate. However, the elution strength of acetonitrile proved excessive, leading to co-elution of lepidilines. Consequently, subsequent trials utilized methanol to address this issue. In later trials, lepidiline A was obtained, albeit contaminated with an unknown compound at a low concentration. The experiment duration was significantly extended; however, using a methanol/acetonitrile mixture (8:2 *v*/*v*) gradient in water was the most effective solution for resolving co-elution issues. This approach successfully mitigated contamination problems and reduced analysis time without compromising the overall separation of lepidilines. The subsequent challenge involved separating lepidilines B and C, which co-eluted despite modifications to the elution system. Previous literature has indicated that incorporating modifiers, such as formic acid or ammonium acetate, can enhance the separation of imidazole alkaloids in liquid chromatography [6,15]. Various concentrations (0.1–0.4%) of formic acid were tested in both solvents (A—methanol: acetonitrile, B—water), along with the addition of ammonium acetate to solvent B (at concentrations ranging from 5 mM to 40 mM). Ultimately, acidifying both solvents with formic acid to 0.2% and incorporating 5 mM ammonium acetate into solvent B effectively facilitated the separation of all four lepidilines. Concentrations of ammonium acetate below 5 mM did not impact the lepidiline separation process. In contrast, increased concentrations (above 5 mM) resulted in longer retention times for all lepidilines without improving the separation of lepidilines B and C.

The successful isolation of sufficient quantities of lepidilines B, C, and D using the CPC technique enabled subsequent bioactivity studies. The anticancer potential of lepidilines B, C, and D was assessed against neuroblastoma cell lines SK-N-SH and SK-N-AS. The incidence of nervous system cancers has risen significantly in recent years, with neuroblastoma representing one of the most prevalent extracranial solid tumors, accounting for approximately 8–10% of all malignant cancers in children. In 90% of cases, tumor symptoms manifest before the age of five [31]. Effective therapies for this cancer remain limited, underscoring the urgent need for enhanced efficacy of existing treatments and the development of new therapeutic strategies for neuroblastoma, as well as the exploration of effective drugs that inhibit alterations in the nervous system. Synthetic Analogs of lepidilines B, C, and D have demonstrated anticancer activity in breast (MCF7 cell line), cervical (HeLa cell line), lung (A549 cell line), liver (HepG2 cell line), and leukemia (HL-60 cell line) models [11]. Unfortunately, reports on the effects of lepidilines B, C, and D specifically on nervous system cancer cells, including neuroblastoma, remain scarce. In this study, all lepidilines tested individually exhibited dose-dependent inhibition of cell viability in both analyzed cell lines, with cytotoxic effects being more pronounced in SK-N-AS cells compared to SK-N-SH cells. Notably, lepidiline B displayed the most potent cytotoxic activity among all tested compounds, with IC50 values of 25.73 µg/mL for SK-N-SH cells and 14.85 µg/mL for SK-N-AS cells. In contrast, lepidiline C demonstrated the weakest activity, with IC50 values of 52.07 µg/mL for SK-N-SH cells and 44.95 µg/mL for SK-N-AS cells. The cytotoxic potential of lepidilines, expressed as IC_50_ values ranging between 15–50 µg/mL in SK-N-SH and SK-N-AS neuroblastoma cell lines, corresponds approximately to 50–200 µM, depending on its molecular weight. In SK-N-SH and SK-N-AS neuroblastoma cell lines, conventional chemotherapeutic agents, including doxorubicin, cisplatin, and etoposide, typically exhibit cytotoxic activity at low micromolar to sub-micromolar concentrations [32,33,34]. Doxorubicin, in particular, demonstrates pronounced potency, with reported IC_50_ values ranging from 0.03 to 1 µM. In contrast, cisplatin and etoposide display IC_50_ values within the 1–30 µM range, depending on the specific cellular context and experimental parameters. IC_50_ values observed for lepidilines suggest markedly lower intrinsic cytotoxic potency. Nonetheless, such compounds may still warrant pharmacological interest, particularly if subsequent investigations in in vivo models reveal preferential cytotoxicity toward malignant cells. If lepidilines demonstrate selective cytotoxicity toward tumor cells with limited toxicity to normal cells, it could still represent a promising lead compound. Moreover, lepidilines may offer synergistic effects in combination with existing chemotherapeutics, potentially allowing for reduced dosages and minimized side effects of conventional drugs. These observations underscore the potential utility of lepidilines as lead structures, meriting further optimization of their chemical scaffold or formulation strategies to enhance their therapeutic efficacy in oncological applications.

The cytotoxicity of all lepidilines was greater in SK-N-AS cells compared to SK-N-SH cells. As mentioned above, lepidiline B exhibited the highest sensitivity across both lines (IC50 = 25.75 µg/mL for SK-N-SH and IC50 = 14.85 µg/mL for SK-N-AS), which was followed by lepidiline D. In contrast, lepidiline C was distinct from the others with significantly lower IC50 values. These differences in the potential may undoubtedly be attributed to the differences in their chemical structure.

Lepidilin A lacks either a methyl or a methoxy group on the imidazole ring, potentially limiting its interactions with target molecules. In contrast, lepidiline B contains a methyl group, which increases its lipophilicity and membrane permeability, which correlates with its pronounced anticancer activity (IC50 = 14.85 µg/mL). Lepidilin C, which contains a methoxyl group, can alter its polarity and receptor interactions but shows reduced efficacy (IC50 = 52.07 µg/mL), possibly connected to its elevated polarity. In the end, lepidiline D, which possesses both methyl and methoxy substituents, keeps the potential attributed to the presence of the methyl group; however, the presence of a methoxyl moiety decreases the total activity so that it does not equal the potency of lepidiline B (IC50 = 30.54 µg/mL).

Functional groups such as methyl and methoxyl substituents are crucial in modulating the biological activity of these compounds, which has been confirmed in the aforementioned studies and in the studies on other alkaloids. This was the case of N-methyllevamisol, which was found to be a more active anti-angiogenic drug than levamisol [35], or the case where the methyl-substituted compounds at the 4-position of the indole ring were found to exhibit more substantial anticancer potential than the non-substituted compounds [36]. Different information can be found on the role of a methoxy group substituted on alkaloids. Some of the studies, however, underline a decreased anticancer potential of a methoxy-substituted drug, which was the case of the study of Vasamsetti et al. [37] who demonstrated a lesser activity of a 4-methoxy substituted pyridine-1,2,4-oxodiazole compounds towards the PC3, A549, DU-145, and MCF-7 cell lines. In this study, a methoxy group may also play the role of a weakening substituent in the studies on the anticancer potential.

The confirmation of the antiproliferative properties of lepidilines B, C, and D highlights their potential for future application within the pharmaceutical industry, either as standalone drugs or as components of pharmaceutical formulations for the treatment of patients with nervous system cancers, including neuroblastoma. However, this necessitates further mechanistic studies conducted in both in vitro and in vivo models to determine their complete safety profile, including their mutagenic or genotoxic potential. The so far published studies in cell lines do not reveal toxicity towards normal cells [9] nor mutagenicity or irritant effects in in silico studies [38]; nevertheless, this information should be confronted with practical laboratory data and using in vivo models.

## 4. Materials and Methods

### 4.1. Materials

The study utilized red phenotypes of *Lepidium meyenii* that were commercially available and purchased in a local herbal shop in Lublin, Poland, in 2021 (producer: BrainMax Pure Maca Powder). A voucher specimen is kept in the Department of Pharmacognosy with the Medicinal Plants Garden at the Medical University of Lublin with the number WK0052021.

The laboratory equipment used throughout the study was described in the Supplementary File.

### 4.2. High-Performance Liquid Chromatography Coupled with Mass Spectrometry (HPLC-ESI-QTOF-MS/MS) Based Analysis of Extracts, Fractions, and Isolates from Lepidium meyenii

The total extract from *Lepidium meyenii* and the resulting fractions were analyzed using the HPLC-ESI-QTOF-MS/MS platform by Agilent Technologies (Santa Clara, CA, USA) composed of a binary pump (G1312C), a degasser (G1322A), an autosampler (G1329B), a PDA detector (G1315D), an ESI-QTOF-MS/MS detector (G6530B) with a Zorbax Eclipse Plus RP-18 chromatographic column (3.5 μm; 150 mm × 2.1 mm) (Agilent Technologies, Santa Clara, CA, USA).

During the analysis, the HPLC thermostat was maintained at 25 °C, and the UV detection was set at 254, 280, 320, and 365 nm. The PDA detector operated in a 190–600 nm wavelength range. Chromatographic separation was achieved with a 10 μL injection volume and a 0.2 mL/min flow rate during a 36-min gradient elution program. The mobile phase consisted of eluent A (0.1% formic acid in water, *v*/*v*) and eluent B (acetonitrile with 0.1% *v*/*v* formic acid). The gradient elution was as follows: 0 min, 1% B; 2 min, 20% B; 3 min, 30% B; 10 min, 40% B; 17 min, 95% B; 19 min, 95% B; 19.5–36 min, 1% B.

Mass spectrometer measurements were conducted under the following conditions: gas temperature was 275 °C and sheath gas temperature was 325 °C; gas flow rate was 12 L/min; capillary voltage was 3000 V; fragmentation voltage was 110 V; collision energy was 10 and 20 V; skimmer voltage was 65 V; and atomizer pressure was 35 psig. In positive ionization mode, the spectra were scanned in the *m*/*z* range of 40–1200 Da to visualize lepidilines. Two of the most intense signals in the total ion chromatogram (TIC) spectrum were automatically fragmented (data-dependent acquisition mode—DDA) to generate their MS/MS spectra in each scan. After acquiring two spectra for a given *m*/*z* value, the selected molecular feature was excluded from further fragmentation for the next 0.2 min.

For the quantitative analysis of the lepidilines content in the studied extracts, the lepidiline A standard purchased from MedChemExpress (CAS No.: 596093-98-0) (Monmouth Junction, NJ, USA) was used (>95% pure by HPLC-MS). Based on the prepared 5 solutions and the drawn calibration curve, the content of the remaining lepidilines was calculated in each extract, giving the concentration of lepidiline A, B, C, and D that was calculated as the lepidiline A equivalent. This procedure was implemented as the standards of the remaining lepidilines have not been commercially available. On the other hand, all compounds belong to the same class and differ from one another only slightly, which brings similar behavior in the chromatographic conditions for all lepidilines.

Several validation parameters were determined to checkthe accuracy of the method. First, the standard of lepidiline A was injected at a series of concentrations between 0.001 and 1 mg/mL, and the linearity range was determined as 0.001–0.1 mg/mL for the standard solution injected at a volume of 10 µL. The registered calibration equation was y = 34,476,132,820.75x + 379,879,111.92, whereas the R^2^ value was 0.9979. The limit of detection (LOD) value, recognized as the smallest measured concentration of the standard, was calculated as 0.22 µg/mL for lepidiline A, whereas the LOQ was determined as 0.66 µg/mL, taking into consideration the fact that LOQ equals three times the LOD value.

### 4.3. Extraction Methods for the Recovery of Specialized Metabolites from Maca

#### 4.3.1. Ultrasonic Bath Extraction

Root powder (250 g) from the red variety of *Lepidium meyenii* was triple extracted (three 30 min cycles) with 600 mL of 80% methanol. The obtained extracts were filtered through filter paper and evaporated using a vacuum evaporator at 45 °C. The dry extract was stored at −18 °C.

#### 4.3.2. Accelerated Solvent Extraction

One gram of plant material was transferred each time to a 34-mL-volume steel extraction cell using a solution of 50% ethanol, 20 s purge time, and 40% flush volume, and various temperatures (50 °C, 60 °C, 70 °C, 80 °C, 90 °C and 100 °C) and extraction cycle number (1, 2 and 3 cycles) settings. The particular polarity of the extract was selected based on the formerly published manuscripts [39]. In the temperature range of 50–80 °C, 2 g of sand was added per sample to minimize the hardening of plant material. In turn, in the temperature range of 90–100 °C, 3 g of sand was used. For each tested temperature, the procedure below was used analogously. The extraction cycles for each tested temperature included a single, double, and triple extraction of 5 min each and one extraction cycle of 15 min. The obtained extracts were evaporated to dryness at 45 °C using a rotary vacuum evaporator. As a result of this step, twenty-four extracts were obtained, and they were directed to the quantitative determination of lepidilines content.

#### 4.3.3. Soxhlet Extraction

Three portions of 10 g of the powdered plant material were weighed, packed into paper containers, and closed with cotton. Three different protocols were run on every sample, including subsequent extraction of the same plant material. The heating level was set at 18, the rinse time was 10 min, and the solvent volume used for extraction was 120 mL. The first extract was prepared by consecutive extraction of maca tubers with hexane, dichloromethane, and methanol (called HDM), using ten cycles for every solvent. The second extract was obtained using hexane, ethyl acetate, and methanol (called HEM), whereas the last one was obtained using hexane, acetone, and methanol (called HAM). As a result, nine extracts were obtained for further analysis. After the extraction process, the instrument was cooled down, and the extracts were evaporated to dryness on a rotary evaporator at 35 °C and stored in a fridge until the analysis.

#### 4.3.4. Supercritical CO_2_ Extraction

The extraction procedure was performed in two separate experiments on a sample of 30 g of powdered maca tubers. The plant material was transferred to a stainless-steel extraction basket placed in the 1 L tank and underwent extraction at 45 °C (the extraction cell’s temperature and the separators’ temperature) with a flow rate of CO_2_ of 25 g/min flow rate. In the first extraction protocol, the pressure was sustained at 150 bar. The extraction was performed using supercritical CO_2_ for 45 min. The extraction was a dynamic process as it employed continuous solvent recycling. After this time, the first batch of the extract (ELM1a) was collected, and the extraction procedure was continued for the following 1 h on the same plant material with 5% of isopropanol that was added as the co-solvent (1.58 mL/min) to increase the polarity of the extraction mixture (ELM1b).

Another extraction protocol performed on a similar batch of 30 g (ELM2a) of the plant material was carried out with CO_2_ at 45 °C at 250 bars for the first 45 min. After this stage, the extract was collected, and the extraction was carried on with a similar gradient of isopropanol (5%, 1.58 mL/min) for the following 1 h (ELM2b).

The liquid extracts from the second stages of both extractions were evaporated to dryness on a rotary evaporator at 45 °C. They were kept in a fridge until the chromatographic analysis but no longer than two weeks. As a result, four extracts were obtained for further study.

### 4.4. Preparation of Fraction Enriched in Lepidilines

**Figure 11:**
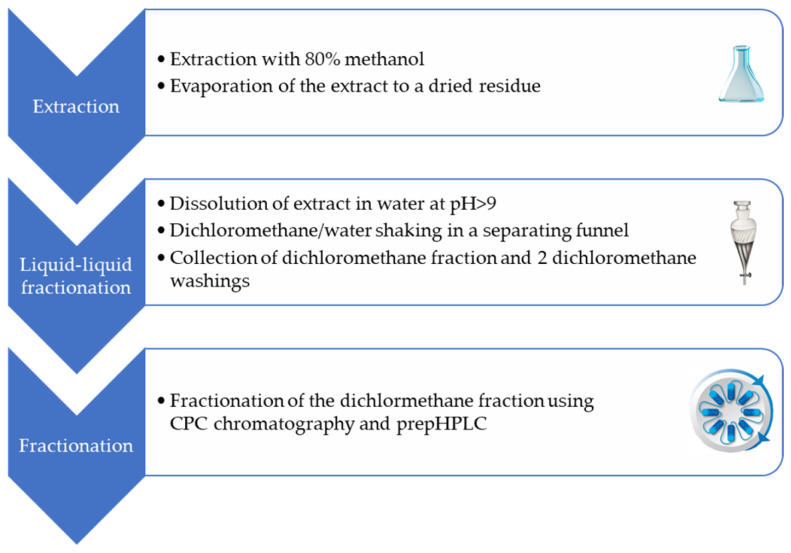
Stages of lepidilines’ purification from the total extract of powdered red maca tuber.

The dry 80% methanol extract (92.1 g) from the red phenotype of *Lepidium meyenii*, obtained through ultrasonic bath extraction, was dissolved in water and then adjusted to a pH > 9 with concentrated ammonia. Then, the solution was transferred to a separating funnel with an equal volume of dichloromethane. After shaking for about 5 min, the upper phase with dichloromethane was removed, whereas the aqueous phase was washed two times more with a fresh portion of dichloromethane. The collected dichloromethane phases were combined and evaporated to dryness using a vacuum rotary evaporator at 40 °C, yielding a 3.4 g lepidilines-rich fraction that was further directed to CPC-based fractionation. The protocol is summarized in the Figure 11 below.

### 4.5. Isolation of Lepidilines by Centrifugal Partition Chromatography (CPC)

#### 4.5.1. Selection and Preparation of the Biphasic System

To select an appropriate biphasic liquid system (BLS), a set of test tubes was prepared that contained 5 mL of differently volumed mixtures of chloroform, methanol, and water, with the addition of 60 mM concentrated hydrochloric acid each. Then, fifty mg of the extract was added to every test tube and vortexed until clear solutions were obtained—the settling time after mixing was measured. After the phases were separated, the aliquots of 500 µL of the lower and upper phases were taken for further analysis on the LC-MS platform. The analysis of the peak areas of respective components in the upper and lower phases was used to calculate the partition coefficient values (K_D_) expressed as the peak area in the upper phase divided by the peak area in the lower phase. Also, K_D_ was calculated for lepidilines concerning the affinity for the lower (L) and upper (U) phases. For the fractionation of the total extract on the CPC apparatus, the selected two-phase system with the desired differences in K_D_ lepidilines was prepared in a separatory funnel with the addition of 60 mM HCl. After vigorously shaking and stabilizing the immiscible liquid system, the two phases were separated into two bottles and used as the upper and lower phases during the fractionation process.

#### 4.5.2. Fractionation Procedure of the Lepidilines-Rich Fraction by CPC

Lepidilines’ isolation was performed using the following protocol: The biphasic solvent system prepared for the experiment was composed of chloroform/methanol/water (4/3/2 *v*/*v*/*v*) with the addition of 60 mM of concentrated HCl. The CPC experiment was conducted in an ascending mode using a heavier (lower) phase as the stationary one that fills the column at first, and a lighter one was injected on a rotating column as the mobile phase, together with the sample that was dissolved in a 50:50 (*v*/*v*) mixture of the upper and lower phases. The stationary phase was pumped into a column rotating at 500 RPM at a flow rate of 20 mL/min for 20 min. Then, the column rotation speed was increased to 1200 RPM, and the upper phase (mobile phase) was pumped at a 4 mL/min flow. After 10 min, 3 g of dissolved extract was injected into the 10-mL injection loop. Throughout the analysis, 5 mL fractions were collected, with the DAD detector set at 230 and 274 nm. From 180 min into the analysis, the flow rate was increased to 8 mL/min, and extrusion with the stationary phase was started and sustained until the end of the analysis, which was at 348 min.

### 4.6. Final Purification of the Isolates by Semi-Preparative High-Performance Liquid Chromatography (HPLC)

The solvents prepared for the analysis, solvent A (water with 5 mM ammonium acetate) and solvent B (methanol: acetonitrile 8:2 *v*/*v*), were both acidified to a concentration of 0.2% (*v*/*v*) with formic acid. The following gradient of the solvent B in A was applied: 0 min 29% B, 85 min 30% B, 120 min 35% B, and 130 min 100% B. The chromatogram was observed at the wavelengths of λ = 205 and 278 nm, and the flow rate was set at 20 mL/min.

The isolated lepidilines were purified from ammonium acetate and formic acid using the following procedure: formic acid was removed through repeated evaporation with methanol, while ammonium acetate was eliminated by dissolving the lepidilines in acetonitrile, a solvent in which lepidilines are soluble but ammonium acetate is not. Then, it was centrifuged, and the supernatant was collected and evaporated to dryness.

### 4.7. Cell Lines

Both human cancer cell lines SK-N-SH (ATCC^®^ HTB-11™) and SK-N-AS (ATCC^®^ CRL-2137™) were grown in DMEM/F12 culture medium supplemented with 10% FBS, penicillin (100 IU/mL), and streptomycin (100 μg/mL) at 37 °C in a humidified atmosphere with 5% CO_2_. All cell lines used in this study were obtained from recognized repositories and handled in accordance with institutional guidelines and relevant national regulations.

### 4.8. Cell Viability Assay

SK-N-SH and SK-N-AS cancer cells were plated on 96-well plates at a 2 × 10^4^ cells/mL density. The cells were incubated with lepidiline B, C, and D at 5–100 µg/mL concentrations for 96 h. Then, the cancer cells were incubated with the MTT (3-(4,5-dimethylthiazol-2-yl)-2,5-diphenyltetrazolium bromide) solution (5 mg/mL, Sigma, St. Louis, MO, USA) for 3 h. During this time, MTT was metabolized by living cells to purple formazan crystals, which were later solubilized in SDS buffer (10% SDS in 0.01 N HCl) overnight. The optical density of the product was measured at 570 nm.

### 4.9. Statistical Analysis

The results were analyzed using GraphPad Prism 5.0 with one-way ANOVA and Tukey’s post-hoc testing. All the data were depicted as the means ± standard deviation (±SD). Results were statistically relevant if *p* < 0.05 (* *p* < 0.05, ** *p* < 0.01, *** *p* < 0.001).

## 5. Conclusions

The manuscript aimed to draw attention to lepidilines—the underestimated constituents of *Lepidium meyenii*. In a few studies, these imidazole alkaloids exhibited anticancer, hormone-regulating, fertility-enhancing, and anti-inflammatory properties. The lack of effective methods for their isolation from plant material encouraged the authors to plan an efficient extraction protocol and deliver precise analytical conditions for their isolation from the total extract.

The performed studies showed a marked role of the solvent type, extraction technique, and temperature on the content of lepidilines in the final maca extract. Organic solvents, like dichloromethane, acetone, or even ethyl acetate, were found to recover these imidazole alkaloids from the plant matrix most efficiently. Also, the elevated temperature increased the efficiency of lepidiline extraction. These parameters were fulfilled when the extraction from powdered maca tubers was performed using Soxhlet extraction. The percentage content of the sum of lepidilines A–D in the dichloromethane extract from the Soxhlet apparatus was calculated as 10.24%.

The conducted extraction delivered an extract rich in several groups of secondary metabolites. To successfully isolate lepidilines from this rich mixture, the CPC chromatography operated in the ascending mode with chloroform/methanol/water (*v*/*v*/*v*) as a biphasic solvent system was used, which followed by the RP semi-preparative HPLC in the gradient of the solvent B in A: 0 min 29% B, 85 min 30% B, 120 min 35% B, and 130 min 100% B.

Although the scientific literature presents a limited number of examples regarding the recovery of lepidilines from extracts, previous studies have demonstrated traditional isolation methods. The protocols involved liquid-liquid extraction and precipitation to separate lepidilines, or a multi-step recovery process that included converting alkaloids into salt forms and utilizing microporous resins for their isolation, but this approach was time-consuming and resulted in considerable sample loss.

In contrast, the application of CPC chromatography combined with preparative high-performance liquid chromatography presented in this study represents a novel and advantageous solution for the isolation of lepidilines from *Lepidium peruvianum*. CPC offers numerous benefits, including the absence of a solid stationary phase, which minimizes the risk of irreversible adsorption, enhances recovery rates, is characterized by low operating costs, and enables selective, repetitive, and high-throughput fractionations. This innovative approach should be considered for industrial-scale isolation of lepidilines, as it enables high sample loadings and significantly improves yields for bioactivity studies.

This work analyzes various solvent systems that can be employed in the fractionation of lepidilines, further supporting the viability of CPC-prepHPLC as a superior method for their efficient recovery. Thanks to the selected techniques, lepidilines B, C, and D were obtained in a quantity that was sufficient for in vitro tests of the anticancer and cytotoxic activity in cell lines. In the study, all lepidilines tested individually demonstrated a dose-dependent inhibition of cell viability in both analyzed cell lines. The cytotoxic effects were more pronounced in SK-N-AS cells compared to SK-N-SH cells. Notably, lepidiline B exhibited the strongest cytotoxic activity among all tested types of compounds.

The extraction and separation conditions elaborated for the sake of this study promise higher recoveries of lepidilines from maca tubers and open the door to their more detailed pharmacological studies. Still, the scientific literature lacks information about their properties delivered from in vivo studies, as well as information on their mechanisms of action and safety. A more efficient recovery of these compounds will certainly help in the continuation of the studies on these interesting compounds of natural origin.

## 6. Patents

The data presented in this manuscript are included in the patent applications P. 447258 and P.447516 submitted to the Patent Office of the Republic of Poland on 22 December 2023 and 16 January 2024, respectively. The titles of the patent applications are “Application of lepidilines in the treatment of nervous system cancer,” and “Method of isolating lepidilines from the extract of maca (*Lepidium meyenii* Walp. syn. *Lepidium peruvianum* Chacon)”.

## Figures and Tables

**Table 1:** Identification data for isolated and purified lepidilines from the red variety of *Lepidium meyenii* recorded in the positive ionization mode. (Ion—ionization mode: positive, delta—error of mass measurement, RDB—rings and double bond number, Rt—retention time, MS/MS fragments in bold represent the ions with the largest intensity).

Rt [min]	Molecular Formula	*m*/*z* Calculated	*m*/*z* Experimental	Delta	RDB	MS/MS Fragments	Identified Compound	References
14.75	C_19_H_20_N_2_	277.1699	277.1701	−0.63	11	198.9383, **185.1093**, 160.1116, 109.0759, **91.0550**	Lepidiline A	[7,9,10,11]
15.33	C_20_H_22_N_2_	291.1856	291.1859	−1.12	11	**250.1615**, **199.1231**, 181.1057, 158.0992, 118.0771, **91.0555**	Lepidiline B	
15.49	C_20_H_22_N_2_O	307.1805	307.1813	−2.65	11	292.1581, 229.1309, 215.1159, **185.1083**, **121.0654**, 106.0414, 91.0539	Lepidiline C	
15.99	C_21_H_24_N_2_O	321.1961	321.1966	−1.44	11	306.1702, 280.1677, 230.1391, **199.1274**, 175.1110, 158.0965, **121.0653**, 91.0541	Lepidiline D	

**Table 2:** The red maca extract’s KD values (lower phase/upper phase) were obtained from different solvent systems.

Compound Name	Biphasic Solvent System Chloroform/Methanol/Water (*v*/*v*/*v*) with HCl Addition							
	4:1.5:2 60 mM HCl	4:2:2 60 mM HCl	4:3:2 60 mM HCl	4:3:3			4:4:2 60 mM HCl	5:5:3 60 mM HCl
				30 mM HCl	60 mM HCl	90 mM HCl		
Lepidiline A	0.63	1.26	2.29	0.66	1.37	1.04	3.09	3.17
Lepidiline B	0.75	1.34	2.76	0.77	1.54	1.25	3.7	4.02
Lepidiline C	0.93	1.62	2.45	0.87	1.64	1.4	3.6	3.86
Lepidiline D	1.08	2.09	4.49	1.09	2.53	1.74	5.37	6.42
Lepidiline E	0.08	0.19	0.85	0.15	0.25	0.25	1.74	1.30

**Table 3:** IC_50_ values obtained in the MTT assay for SK-N-SH (ATCC^®^ HTB-11™) and SK-N-AS (ATCC^®^ CRL-2137™) human neuroblastoma cell lines treated with lepidiline B, C, and D for 96 h. The data are presented as the mean ± standard error (±SE).

[µg/mL]	SK-N-SH	SK-N-AS
Lepidiline B	25.73 ± 0.051	14.85 ± 0.079
Lepidiline C	52.07 ± 0.032	44.95 ± 0.033
Lepidiline D	30.54 ± 0.040	17.09 ± 0.083

## Data Availability

All data are presented in the manuscript and Supplementary File.

## Supplementary Materials

The following supporting information can be downloaded at https://www.mdpi.com/article/10.3390/molecules30224360/s1: Table S1: The list of solvents taken for system evaluation; Table S2: The quantification of lepidilines in ASE extracts; Table S3: The SD values of the results obtained in ASE extraction; Table S4. The quantification of lepidilines in Soxhlet extracts; Table S5. The SD values of the results obtained in the Soxhlet apparatus, Table S6. The quantification of lepidilines in SFE extracts; Table S7. Standard deviation values from the results obtained in SFE extraction; Table S8. Differences in statistical significance between extracts 1–37 obtained using different extraction methods. *n* = 3 from three independent experiments. * *p*  <  0.05, ** *p*  <  0.01, *** *p*  <  0.001 by one-way ANOVA, Tukey’s post-hoc test (the coding used is explained in the Table S9); Table S9. The coding of the obtained extracts used in Table S8—the used extraction parameters with the given extract number; Figure S1. % of lepidilines A–E in samples 1 (A), 2 (B), 3 (C), 4 (D), 5 (E), 6 (F). *n* = 3 from 3 independent experiments; Figure S2. % of lepidilines A–E in samples 7 (A), 8 (B), 9 (C), 10 (D), 11 (E), 12 (F). *n* = 3 from 3 independent experiments; Figure S3. % of lepidilines A–E in samples 13 (A), 14 (B), 15 (C), 16(D), 17(E), 18(F). *n* = 3 from 3 independent experiments; Figure S4. % of lepidilines A–E in samples 19 (A), 20 (B), 21 (C), 22 (D), 23 (E), 24 (F). *n* = 3 from 3 independent experiments; Figure S5. % of lepidilines A–E in samples 25 (A), 26 (B), 27 (C), 28 (D), 29 (E), 30 (F). *n* = 3 from 3 independent experiments; Figure S6. % of lepidilines A–E in samples 31 (A), 32 (B), 33 (C), 34 (D), 35 (E), 36 (F), 37 (G). *n* = 3 from 3 independent experiments. Figure S7. LC/MS (ESI+) MS/MS spectra of the Lepidiline A, B, C, and D.

## References

[1] Ahmad R., Ahmad N., Al-Anaki W.S., Ismail F.A., Al-Jishi F. (2020). Solvent and Temperature Effect of Accelerated Solvent Extraction (ASE) Coupled with Ultra-High-Pressure Liquid Chromatography (UHPLC-PDA) for the Determination of Methyl Xanthines in Commercial Tea and Coffee. Food Chem..

[2] Brooks N.A., Wilcox G., Walker K.Z., Ashton J.F., Cox M.B., Stojanovska L. (2008). Beneficial Effects of Lepidium Meyenii (Maca) on Psychological Symptoms and Measures of Sexual Dysfunction in Postmenopausal Women Are Not Related to Estrogen or Androgen Content. Menopause.

[3] Zenico T., Cicero A.F.G., Valmorri L., Mercuriali M., Bercovich E. (2009). Subjective Effects of *Lepidium Meyenii* (Maca) Extract on Well-Being and Sexual Performances in Patients with Mild Erectile Dysfunction: A Randomised, Double-Blind Clinical Trial. Andrologia.

[4] Ulloa Del Carpio N., Alvarado-Corella D., Quiñones-Laveriano D.M., Araya-Sibaja A., Vega-Baudrit J., Monagas-Juan M., Navarro-Hoyos M., Villar-López M. (2024). Exploring the Chemical and Pharmacological Variability of Lepidium Meyenii: A Comprehensive Review of the Effects of Maca. Front. Pharmacol..

[5] Minich D.M., Ross K., Frame J., Fahoum M., Warner W., Meissner H.O. (2024). Not All Maca Is Created Equal: A Review of Colors, Nutrition, Phytochemicals, and Clinical Uses. Nutrients.

[6] Le H.T.N., Van Roy E., Dendooven E., Peeters L., Theunis M., Foubert K., Pieters L., Tuenter E. (2021). Alkaloids from Lepidium Meyenii (Maca), Structural Revision of Macaridine and UPLC-MS/MS Feature-Based Molecular Networking. Phytochemistry.

[7] Cheng C., Shen F., Ding G., Liu A., Chu S., Ma Y., Hou X., Hao E., Wang X., Hou Y. (2020). Lepidiline A Improves the Balance of Endogenous Sex Hormones and Increases Fecundity by Targeting HSD17B1. Mol. Nutr. Food Res..

[8] Bai N., He K., Roller M., Lai C.-S., Bai L., Pan M.-H. (2015). Flavonolignans and Other Constituents from *Lepidium Meyenii* with Activities in Anti-Inflammation and Human Cancer Cell Lines. J. Agric. Food Chem..

[9] Curran D., Dada O., Müller-Bunz H., Rothemund M., Sánchez-Sanz G., Schobert R., Zhu X., Tacke M. (2018). Synthesis and Cytotoxicity Studies of Novel NHC*-Gold(I) Complexes Derived from Lepidiline A. Molecules.

[10] Curran D., Müller-Bunz H., Bär S.I., Schobert R., Zhu X., Tacke M. (2020). Novel Anticancer NHC*-Gold(I) Complexes Inspired by Lepidiline A. Molecules.

[11] Mlostoń G., Kowalczyk M., Celeda M., Jasiński M., Denel-Bobrowska M., Olejniczak A.B. (2022). Fluorinated Analogues of Lepidilines A and C: Synthesis and Screening of Their Anticancer and Antiviral Activity. Molecules.

[12] Zhou Y., Li P., Brantner A., Wang H., Shu X., Yang J., Si N., Han L., Zhao H., Bian B. (2017). Chemical Profiling Analysis of Maca Using UHPLC-ESI-Orbitrap MS Coupled with UHPLC-ESI-QqQ MS and the Neuroprotective Study on Its Active Ingredients. Sci. Rep..

[13] Tarabasz D., Szczeblewski P., Laskowski T., Płaziński W., Baranowska-Wójcik E., Szwajgier D., Kukula-Koch W., Meissner H.O. (2022). The Distribution of Glucosinolates in Different Phenotypes of Lepidium Peruvianum and Their Role as Acetyl- and Butyrylcholinesterase Inhibitors—In Silico and In Vitro Studies. IJMS.

[14] Szalak R., Matysek M., Koval M., Dziedzic M., Kowalczuk-Vasilev E., Kruk-Slomka M., Koch W., Arciszewski M.B., Kukula-Koch W. (2023). Magnoflorine from Berberis Vulgaris Roots—Impact on Hippocampal Neurons in Mice after Short-Term Exposure. Int. J. Mol. Sci..

[15] Jin W., Chen X., Dai P., Yu L. (2016). Lepidiline C and D: Two New Imidazole Alkaloids from Lepidium Meyenii Walpers (Brassicaceae) Roots. Phytochem. Lett..

[16] Brent Friesen J., Pauli G.F. (2005). G.U.E.S.S.—A Generally Useful Estimate of Solvent Systems for CCC. J. Liq. Chromatogr. Relat. Technol..

[17] Kukula-Koch W., Koch W., Angelis A., Halabalaki M., Aligiannis N. (2016). Application of pH-Zone Refining Hydrostatic Countercurrent Chromatography (hCCC) for the Recovery of Antioxidant Phenolics and the Isolation of Alkaloids from Siberian Barberry Herb. Food Chem..

[18] Solórzano-Acosta R., Chanco M., Seminario M., Cabello-Torres R., Camel V., Lastra S., Arias A., Verástegui P., Quispe K., Carbajal-Llosa C. (2025). Soil Management in Lepidium Meyenii (Maca) Monoculture: Trends and Challenges for Small Farmers around Lake Chinchaycocha in the Andean Highlands of Junin (Peru). Front. Soil. Sci..

[19] Gonzales G.F. (2012). Ethnobiology and Ethnopharmacology of *Lepidium Meyenii* (Maca), a Plant from the Peruvian Highlands. Evid.-Based Complement. Altern. Med..

[20] Huang Y.-J., Peng X.-R., Qiu M.-H. (2018). Progress on the Chemical Constituents Derived from Glucosinolates in Maca (*Lepidium Meyenii*). Nat. Prod. Bioprospect..

[21] Zhou Y., Zhu L., Li H., Xie W., Liu J., Zhang Y., Li Y., Wang C. (2022). In Vivo and in Vitro Neuroprotective Effects of Maca Polysaccharide. Front. Biosci..

[22] Gorgani L., Mohammadi M., Najafpour G.D., Nikzad M. (2017). Sequential Microwave-Ultrasound-Assisted Extraction for Isolation of Piperine from Black Pepper (*Piper Nigrum* L.). Food Bioprocess Technol..

[23] Cui B., Zheng B.L., He K., Zheng Q.Y. (2003). Imidazole Alkaloids from *Lepidium meyenii*. J. Nat. Prod..

[24] Dzięcioł M., Wróblewska A., Janda-Milczarek K. (2023). Comparative Studies of DPPH Radical Scavenging Activity and Content of Bioactive Compounds in Maca (*Lepidium Meyenii*) Root Extracts Obtained by Various Techniques. Appl. Sci..

[25] Chen S.-X., Li K.-K., Pubu D., Jiang S.-P., Chen B., Chen L.-R., Yang Z., Ma C., Gong X.-J. (2017). Optimization of Ultrasound-Assisted Extraction, HPLC and UHPLC-ESI-Q-TOF-MS/MS Analysis of Main Macamides and Macaenes from Maca (Cultivars of Lepidium Meyenii Walp). Molecules.

[26] Berthod A., Ruiz-Ángel M.J., Carda-Broch S. (2009). Countercurrent Chromatography: People and Applications. J. Chromatogr. A.

[27] Bourdat-Deschamps M., Herrenknecht C., Akendengue B., Laurens A., Hocquemiller R. (2004). Separation of Protoberberine Quaternary Alkaloids from a Crude Extract of Enantia Chlorantha by Centrifugal Partition Chromatography. J. Chromatogr. A.

[28] Bojczuk M., Żyżelewicz D., Hodurek P. (2017). Centrifugal Partition Chromatography—A Review of Recent Applications and Some Classic References. J. Sep. Sci..

[29] Yang F., Zhang T., Zhang R., Ito Y. (1998). Application of Analytical and Preparative High-Speed Counter-Current Chromatography for Separation of Alkaloids from Coptis Chinensis Franch. J. Chromatogr. A.

[30] Zwerger M., Boeck L., Manzl J., Schwaiger S., Ganzera M. (2024). Novel Approaches for the Analysis and Isolation of Benzylisoquinoline Alkaloids in Chelidonium Majus. Planta Med..

[31] Heck J.E., Ritz B., Hung R.J., Hashibe M., Boffetta P. (2009). The Epidemiology of Neuroblastoma: A Review. Paediatr. Perinat. Epid..

[32] Lautz T.B., Jie C., Clark S., Naiditch J.A., Jafari N., Qiu Y.-Y., Zheng X., Chu F., Madonna M.B. (2012). The Effect of Vorinostat on the Development of Resistance to Doxorubicin in Neuroblastoma. PLoS ONE.

[33] Xu Z., Sun Y., Wang D., Sun H., Liu X. (2020). SNHG16 Promotes Tumorigenesis and Cisplatin Resistance by Regulating miR-338-3p/PLK4 Pathway in Neuroblastoma Cells. Cancer Cell Int..

[34] Najem S., Langemann D., Appl B., Trochimiuk M., Hundsdoerfer P., Reinshagen K., Eschenburg G. (2016). Smac Mimetic LCL161 Supports Neuroblastoma Chemotherapy in a Drug Class-Dependent Manner and Synergistically Interacts with ALK Inhibitor TAE684 in Cells with ALK Mutation F1174L. Oncotarget.

[35] Ali I., Lone M.N., Aboul-Enein H.Y. (2017). Imidazoles as Potential Anticancer Agents. Med. Chem. Commun..

[36] Sharma P., LaRosa C., Antwi J., Govindarajan R., Werbovetz K.A. (2021). Imidazoles as Potential Anticancer Agents: An Update on Recent Studies. Molecules.

[37] Vasamsetti R., Gatchakayala N.B., Bujjibabu N., Kumar V.S., Satyanarayana T.V.V., Pavani G., Kapavarapu R.K., Madhav B. (2025). Rational Design, and Synthesis of Imidazole Ring Incorporated Pyridine-1,2,4-Oxadiazole Derivatives: In-Vitro Anticancer Evaluation and in-Silico Molecular Docking Simulations. Results Chem..

[38] Ibrahim R.M., Elmasry G.F., Refaey R.H., El-Shiekh R.A. (2022). *Lepidium Meyenii* (Maca) Roots: UPLC-HRMS, Molecular Docking, and Molecular Dynamics. ACS Omega.

[39] Meissner H.O., Mscisz A., Mrozikiewicz M., Baraniak M., Mielcarek S., Kedzia B., Piatkowska E., Jólkowska J., Pisulewski P. (2015). Peruvian Maca (*Lepidium Peruvianum*): (I) Phytochemical and Genetic Differences in Three Maca Phenotypes. Int. J. Biomed. Sci..

