# Supplementary material for: Purification of Lepidilines A, B, C, and D from Lepidium meyenii Walpers by Centrifugal Partition Chromatography Followed by Semi-Preparative HPLC and Preliminary Evaluation of Anticancer Activity Against Neuroblastoma Cell Lines
Source: Molecules. 2025 Nov 11;30(22):4360. doi: 10.3390/molecules30224360 (PMC12655379; doi:10.3390/molecules30224360)

Supplementary File

Purification of Lepidilines A, B, C, and D from Lepidium meyenii Walpers by Centrifugal Partition Chromatography Followed by Semi-Preparative HPLC and Preliminary Evaluation of Anticancer Activity Against Neuroblastoma Cell Lines

Tarabasz Dominik[1,*], Estera Okon[2], Anna Wawruszak[2], Stavros Beteinakis[3], Apostolis Angelis[3], Henry O. Meissner[4,†], Leandros A. Skaltsounis[3], Wirginia Kukula-Koch[1,*]

EQUIPMENT USED IN THE STUDIES

4.2. High-Performance Liquid Chromatography Coupled with Mass Spectrometry (HPLC-ESI-QTOF-MS/MS) based analysis of extracts, fractions and isolates from Lepidium meyenii.

The total extract from *Lepidium meyenii* and the resulting fractions were analyzed using the HPLC-ESI-QTOF-MS/MS platform by Agilent Technologies (Santa Clara, California, USA). The instrument was composed of different modules that included a photodiode array detector (G1315D), a binary pump (G1312C), an autosampler (G1329B), and a degasser (G1322A). As a detector, an ESI-QTOF-MS/MS mass spectrometer (G6530B) was also attached to the HPLC chromatograph (1200 series). During the analysis of the samples, a Zorbax Eclipse Plus RP-18 chromatographic column (3.5 μm; 150 mm × 2.1 mm) by Agilent Technologies was used. The Agilent MassHunter Workstation Software (version B.12.00) was utilized to acquire the MS spectra and process the data.

4.3. Extraction Methods for the Recovery of Specialised Metabolites from Maca

4.3.1. Ultrasonic Bath Extraction

The extraction of red maca powder was achieved with an ultrasonic device type RK 510 H (Bandelin SONOREX, Berlin, Germany) operating at a high frequency of 35 kHz.

4.3.2. Accelerated Solvent Extraction

The ASE extraction was performed using an ASE 100 extractor (Dionex, Sunnyvale, USA) on a powdered plant material.

4.3.3. Soxhlet Extraction

The universal Extractor E-800 (Buchi, Flawil, Sankt Gallen, Switzerland) prepared extracts of different polarities.

4.3.4. Supercritical CO_2 extraction

The CO_2 extraction was performed using the SFE-1-2 No 4218 system (Separex F 54250, Champigneulles, France) in different pressure settings

using carbon dioxide in its supercritical state solely and with a gradient of 5 % isopropanol (IP) to achieve a polarity increase. The instrument is a pilot-scale extractor that is composed of a CO_2 tank, a pump for the CO_2 gas, extraction vessels of 1 and 2 L connected between themselves and also together with three separators of 200 mL each, an additional pump for the co-solvent and a cooling system.

4.5. Isolation of Lepidilines by Centrifugal Partition Chromatography (CPC)

4.5.2. Fractionation Procedure of the Lepidilines-rich Fraction by CPC

The equipment to separate lepidilines from the total extract was produced by Armen company, model SCPC-250-L (France). The CPC system consisted of a column/rotor with a capacity of 250 mL connected to a four-channel pump and equipped with an externally connected detector (DAD 600 Flash 06S) and an automatic fraction collector (LS 5600).

4.6. Final purification of the isolates by semi-preparative High-Performance Liquid Chromatography (HPLC)

The semi-preparative HPLC obtained from Shimadzu Corporation (Kyoto, Japan) that was used in this study was composed of a PDA detector (SPD-M40), a column oven (CTO-40C), a solvent delivery module (LC-20AP), a system controller (CBM-40) and a liquid handler (LH-40). The purification method was optimized on the preparative column ReproSil-Pur 120 C18-AQ (250 mm x 20 mm, 5.0 µm) by Dr. Maisch (Tubingen, Germany).

4.7. Cell Lines

SK-N-SH (ATCC® HTB-11™) and SK-N-AS (ATCC® CRL-2137™) human neuroblastoma cell lines were obtained from the American Type Culture Collection (Manassas, VA, USA). DMEM/F12 culture medium, FBS, penicillin, and streptomycin were all purchased from (Sigma, St. Louis, MO, USA)

4.8. Cell Viability Assay

The optical density of the product was measured at 570 nm using an Infinite M200 Pro microplate reader (Tecan, Männedorf, Switzerland).

Table S1. The list of solvents taken for system evaluation

Hexane	Ethyl acetate	Methanol	Water	Butanol	Methyl-t-Butyl Ether	Acetonitrile	Heptane
8	2	5	5				
3	5	3	5				
1	5	1	5				
2			5	3			
	5		5				
	2		5	3			
			5	5			
	1		5	4			
	3		5	2			
	4		5	2			
	5		4			1	
	4		4			1	
	3		4			1	
	3		3			2	
			5		4	1	
			8		6	3	
			3		2	2	
			8		4	3	
			5		2	1	
			40	44		6	10

Table S2. The quantification of lepidilines in ASE extracts

Temperature [°C]	Number and duration of extraction cycles	Lepidiline A	Lepidiline B	Lepidiline C	Lepidiline D
50°C	1x5 min	0.468%	0.459%	0.079%	0.170%
	2x5 min	0.458%	0.476%	0.079%	0.174%
	3x5 min	0.460%	0.472%	0.078%	0.168%
	1x15 min	0.429%	0.429%	0.072%	0.155%
60°C	1x5 min	0.426%	0.420%	0.070%	0.161%
	2x5 min	0.432%	0.425%	0.071%	0.159%
	3x5 min	0.423%	0.440%	0.070%	0.155%
	1x15 min	0.426%	0.421%	0.069%	0.143%
70°C	1x5 min	0.421%	0.438%	0.072%	0.155%
	2x5 min	0.427%	0.453%	0.075%	0.163%
	3x5 min	0.422%	0.423%	0.072%	0.158%
	1x15 min	0.439%	0.437%	0.078%	0.166%
80°C	1x5 min	0.540%	0.505%	0.093%	0.202%
	2x5 min	0.466%	0.464%	0.080%	0.183%
	3x5 min	0.484%	0.511%	0.084%	0.201%
	1x15 min	0.465%	0.463%	0.080%	0.190%
90°C	1x5 min	0.480%	0.495%	0.084%	0.198%
	2x5 min	0.469%	0.460%	0.080%	0.181%
	3x5 min	0.450%	0.489%	0.081%	0.177%
	1x15 min	0.485%	0.479%	0.086%	0.192%
100°C	1x5 min	0.473%	0.491%	0.084%	0.189%
	2x5 min	0.460%	0.464%	0.082%	0.187%
	3x5 min	0.491%	0.532%	0.092%	0.203%
	1x15 min	0.481%	0.505%	0.085%	0.197%

Table S3. The SD values of the results obtained in ASE extraction

Temperature [°C]	Number and duration of extraction cycles	Lepidiline A	Lepidiline B	Lepidiline C	Lepidiline D
50°C	1x5 min	0.014%	0.051%	0.004%	0.006%
	2x5 min	0.000%	0.028%	0.007%	0.008%
	3x5 min	0.006%	0.019%	0.007%	0.001%
	1x15 min	0.036%	0.070%	0.009%	0.009%
60°C	1x5 min	0.047%	0.055%	0.007%	0.007%
	2x5 min	0.026%	0.039%	0.004%	0.007%
	3x5 min	0.021%	0.005%	0.004%	0.008%
	1x15 min	0.007%	0.016%	0.001%	0.005%
70°C	1x5 min	0.016%	0.004%	0.002%	0.005%
	2x5 min	0.005%	0.022%	0.000%	0.001%
	3x5 min	0.029%	0.035%	0.003%	0.009%
	1x15 min	0.017%	0.047%	0.005%	0.009%
80°C	1x5 min	0.019%	0.062%	0.007%	0.008%
	2x5 min	0.027%	0.040%	0.002%	0.002%
	3x5 min	0.017%	0.005%	0.002%	0.009%
	1x15 min	0.023%	0.053%	0.005%	0.012%
90°C	1x5 min	0.046%	0.038%	0.013%	0.024%
	2x5 min	0.064%	0.099%	0.013%	0.013%
	3x5 min	0.068%	0.084%	0.011%	0.014%
	1x15 min	0.067%	0.055%	0.014%	0.021%
100°C	1x5 min	0.073%	0.083%	0.010%	0.016%
	2x5 min	0.045%	0.052%	0.009%	0.015%
	3x5 min	0.041%	0.061%	0.008%	0.011%
	1x15 min	0.036%	0.059%	0.007%	0.012%

Table S4. The quantification of lepidilines in Soxhlet extracts.

		Lepidiline A	Lepidiline B	Lepidiline C	Lepidiline D
HDM extract	Hexane	0.41%	0.12%	0.17%	0.04%
	DCM	3.21%	2.87%	1.72%	2.44%
	Methanol	1.28%	1.56%	0.38%	0.89%
HEM extract	Hexane	0.89%	0.32%	0.37%	0.15%
	Ethyl acetate	1.74%	1.56%	0.79%	1.18%
	Methanol	1.14%	1.26%	0.31%	0.79%
HAM extract	Hexane	1.28%	0.57%	0.56%	0.31%
	Acetone	2.93%	2.58%	1.37%	2.03%
	Methanol	1.03%	1.06%	0.28%	0.69%
Total Lepidilines value	HDM extract	4.90%	4.54%	2.27%	3.37%
	HEM extract	3.78%	3.13%	1.47%	2.12%
	HAM extract	5.24%	4.21%	2.21%	3.03%

Table S5. The SD values of the results obtained in Soxhlet apparatus

	HDM extract			HEM extract			HAM extract		
	Hexane	DCM	Methanol	Hexane	Ethyl acetate	Methanol	Hexane	Acetone	Methanol
LEA	0.00%	0.04%	0.01%	0.06%	0.04%	0.17%	0.08%	0.05%	0.08%
LEB	0.01%	0.01%	0.02%	0.03%	0.09%	0.04%	0.03%	0.02%	0.02%
LEC	0.01%	0.01%	0.01%	0.09%	0.04%	0.06%	0.02%	0.01%	0.02%
LED	0.00%	0.01%	0.01%	0.04%	0.03%	0.10%	0.02%	0.01%	0.02%

Table S6. The quantification of lepidilines in SFE extracts

	Lepidiline A	Lepidiline B	Lepidiline C	Lepidiline D
ELM1 extract	1.15%	0.13%	0.64%	0.05%
ELM2 extract	1.05%	0.15%	0.70%	0.07%

Table S7. Standard deviation values from the results obtained in SFE extraction

	Lepidiline A	Lepidiline B	Lepidiline C	Lepidiline D
ELM1 extract	0.09%	0.02%	0.03%	0.02%
ELM2 extract	0.01%	0.01%	0.02%	0.00%

Table S8. Differences in statistical significance between extracts 1–37 obtained using different extraction methods. n=3 from three independent experiments. *$p < 0.05$, **$p < 0.01$, ***$p < 0.001$ by one-way ANOVA, Tukey's post-hoc test (the coding used is explained in the Table S9).

	1	2	3	4	5	6	7	8	9	10	11	12	13	14	15	16	17	18	19	20	21	22	23	24	25	26	27	28	29	30	31	32	33	34	35	36	37
1																										*	***	***	***	***	***				***	***	***
2																										**	***	***	***	***	***			***	***	***	***
3																										**	***	***	***	***	***			***	***	***	***
4													*													***	***	***	***	***	***	*	**	***	***	***	***
5													**													***	***	***	***	***	*	**	***	***	***	***	***
6													**													***	***	***	***	***	*	**	***	***	***	***	***
7													**													***	***	***	***	***	*	**	***	***	***	***	***
8													**													***	***	***	***	***	*	**	***	***	***	***	***
9													**													***	***	***	***	***	***	**	**	***	***	***	***
10													**													***	***	***	***	***	***	*	**	***	***	***	***
11													**													***	***	***	***	***	***	*	**	***	***	***	*
12													*													***	***	***	***	***	***		**	***	***	***	*
13																									***		***	***	***	***				***	***	***	***
14																										*	***	***	***	***	***			***	***	***	***
15																									*		***	***	***	***	**			***	***	***	***
16																										*	***	***	***	***	***			***	***	***	***
17																											***	***	***	***	**			***	***	***	***
18																										*	***	***	***	***	***			***	***	***	***
19																										**	***	***	***	***	***			***	***	***	***
20																									*		***	***	***	***	**			***	***	***	***
21																										*	***	***	***	***	***			***	***	***	***
22																										*	***	***	***	***	***			***	***	***	***
23																									*		***	***	***	***	*			***	***	***	***
24																											***	***	***	***	**			***	***	***	***
25																										***	***	***	***	***	***	***	***	***	***	***	***
26																											***	***	***	***				***	***	***	***
27																												***	***	***	***	***	***	***	*	***	***
28																													***	***	***	***	***	***	***	***	***
29																														***	***	***	***	***	***	***	***
30																															***	***	***	***	***	*	***
31																																		***	***	***	***
32																																		***	***	***	***
33																																		***	***	***	***
34																																				***	***
35																																				***	***
36																																					***
37																																					

Table S9. The coding of the obtained extracts used in Table S8 – the used extraction parameters with the given extract number.

Extraction technique	Temperature [°C]	Number and duration of extraction cycles	
ASE	50°C	1x5 min	**1**
		2x5 min	**2**
		3x5 min	**3**
		1x15 min	**4**
	60°C	1x5 min	**5**
		2x5 min	**6**
		3x5 min	**7**
		1x15 min	**8**
	70°C	1x5 min	**9**
		2x5 min	**10**
		3x5 min	**11**
		1x15 min	**12**
	80°C	1x5 min	**13**
		2x5 min	**14**
		3x5 min	**15**
		1x15 min	**16**
	90°C	1x5 min	**17**
		2x5 min	**18**
		3x5 min	**19**
		1x15 min	**20**
	100°C	1x5 min	**21**
		2x5 min	**22**
		3x5 min	**23**
		1x15 min	**24**
SFE	CO2 + 5% isopropanol	150 bar	**34**
	CO2 + 5% isopropanol	250 bar	**35**
	CO2	150 bar	**36**
	CO2	250 bar	**37**
SOXHLET	HDM extract	Hexane	**25**
		DCM	**26**
		Methanol	**27**
	HEM extract	Hexane	**28**
		Ethyl acetate	**29**
		Methanol	**30**
	HAM extract	Hexane	**31**
		Acetone	**32**
		Methanol	**33**

Figure S1. % of lepidilines A-E in samples 1 (A), 2 (B), 3 (C), 4(D), 5(E), 6(F). n=3 from 3 independent experiments.

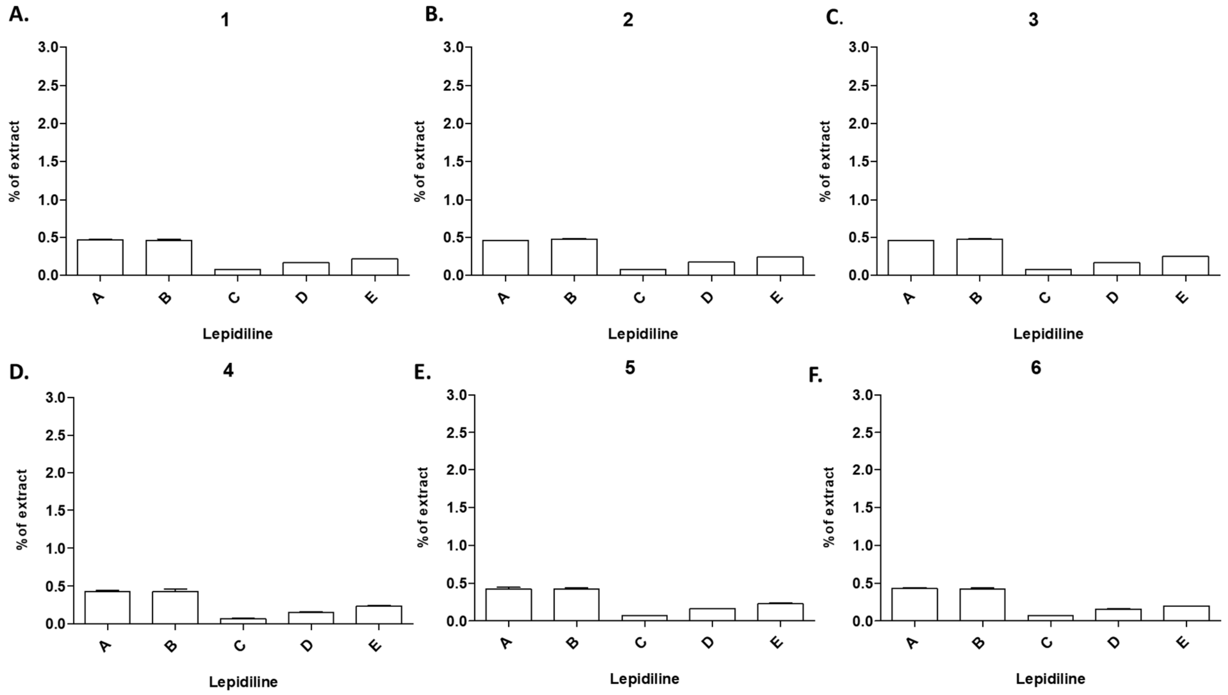

Figure S2. % of lepidilines A-E in samples 7 (A), 8 (B), 9 (C), 10(D), 11(E), 12(F). n=3 from 3 independent experiments.

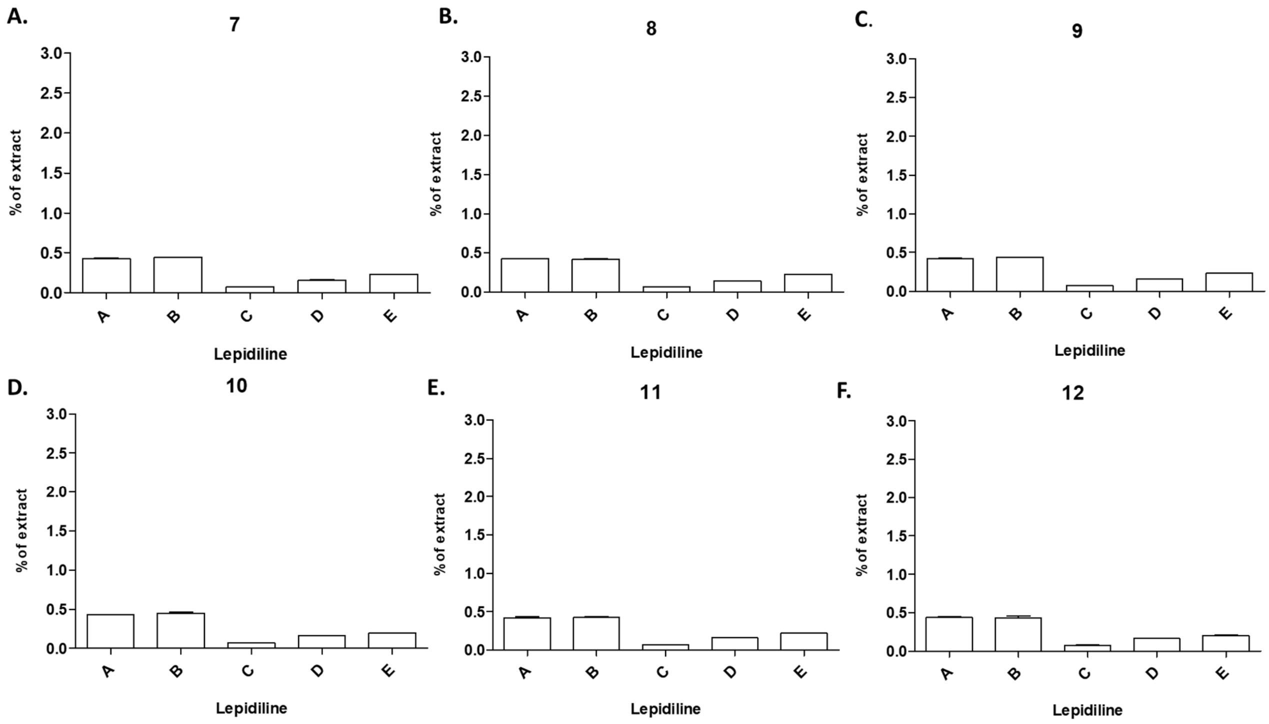

Figure S3. % of lepidilines A-E in samples 13 (A), 14 (B), 15 (C), 16(D), 17(E), 18(F). n=3 from 3 independent experiments.

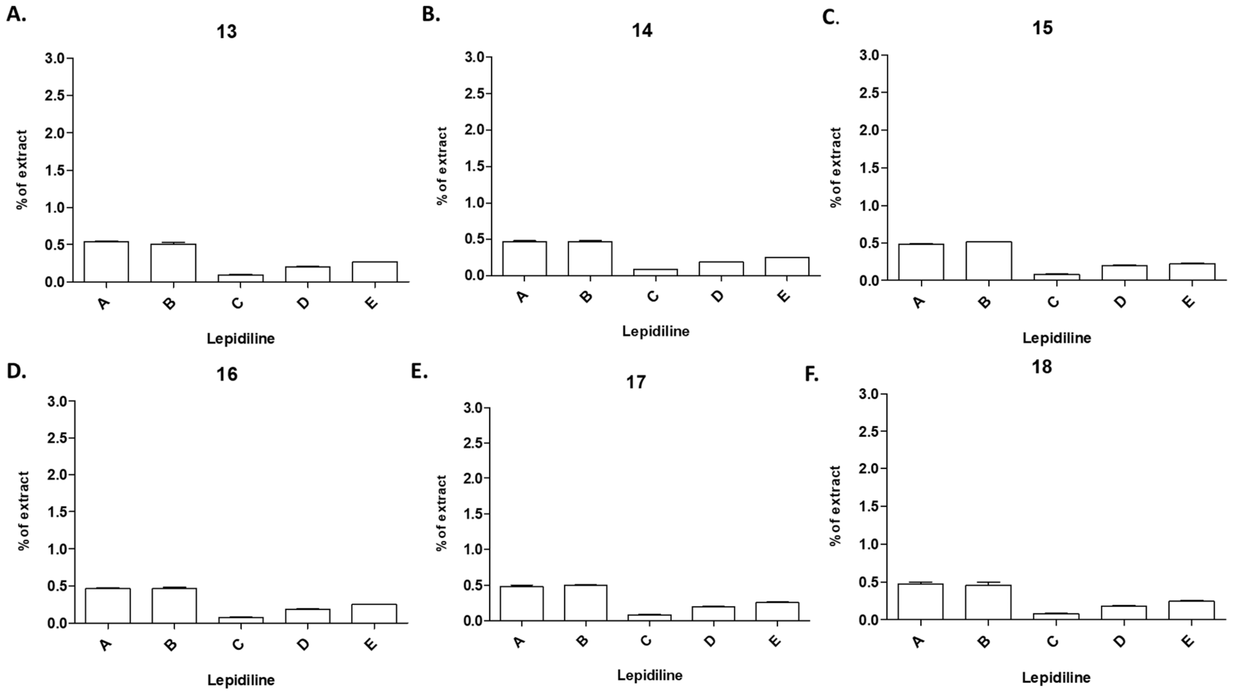

Figure S4. % of lepidilines A-E in samples 19 (A), 20 (B), 21 (C), 22(D), 23(E), 24(F). n=3 from 3 independent experiments.

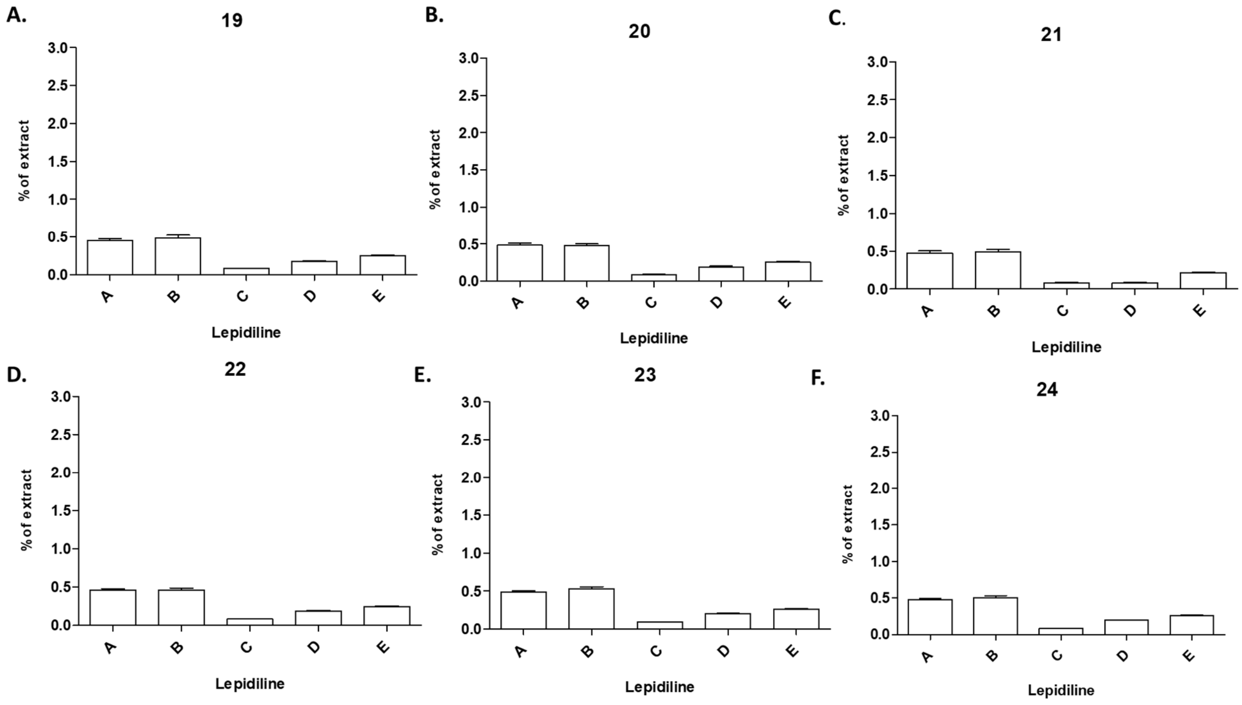

Figure S5. % of lepidilines A-E in samples 25 (A), 26 (B), 27 (C), 28(D), 29(E), 30(F). n=3 from 3 independent experiments.

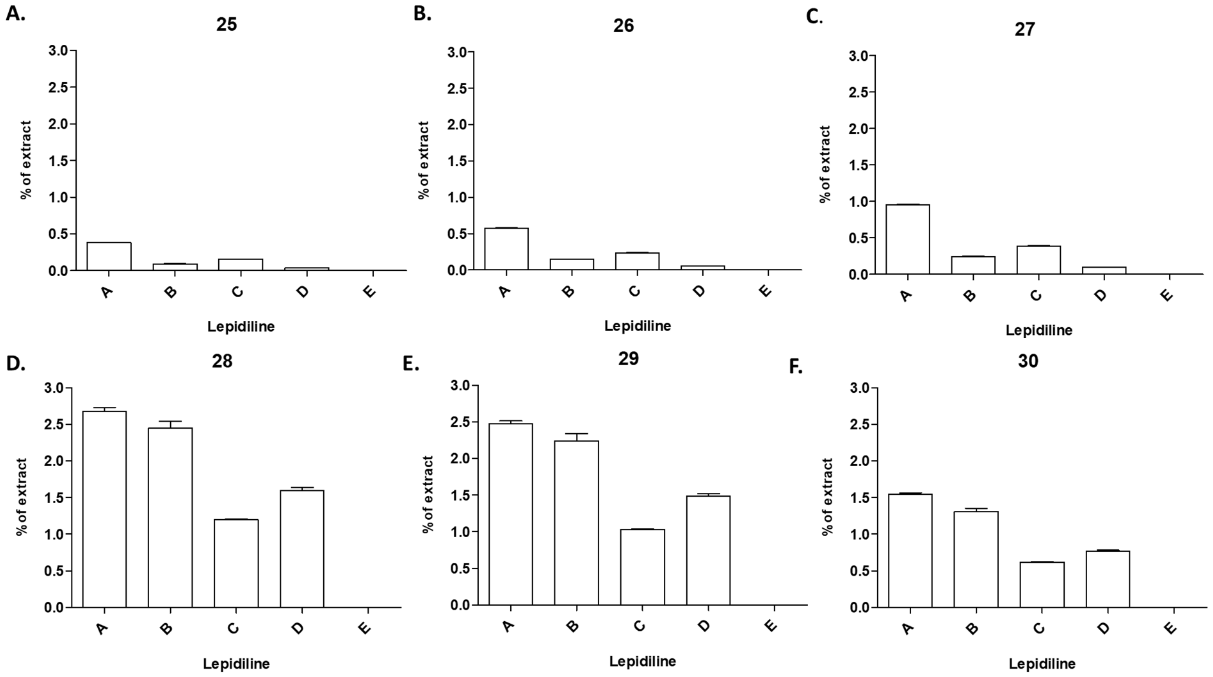

Figure S6. % of lepidilines A-E in samples 31 (A), 32 (B), 33 (C), 34(D), 35(E), 36(F), 37 (G). n=3 from 3 independent experiments.

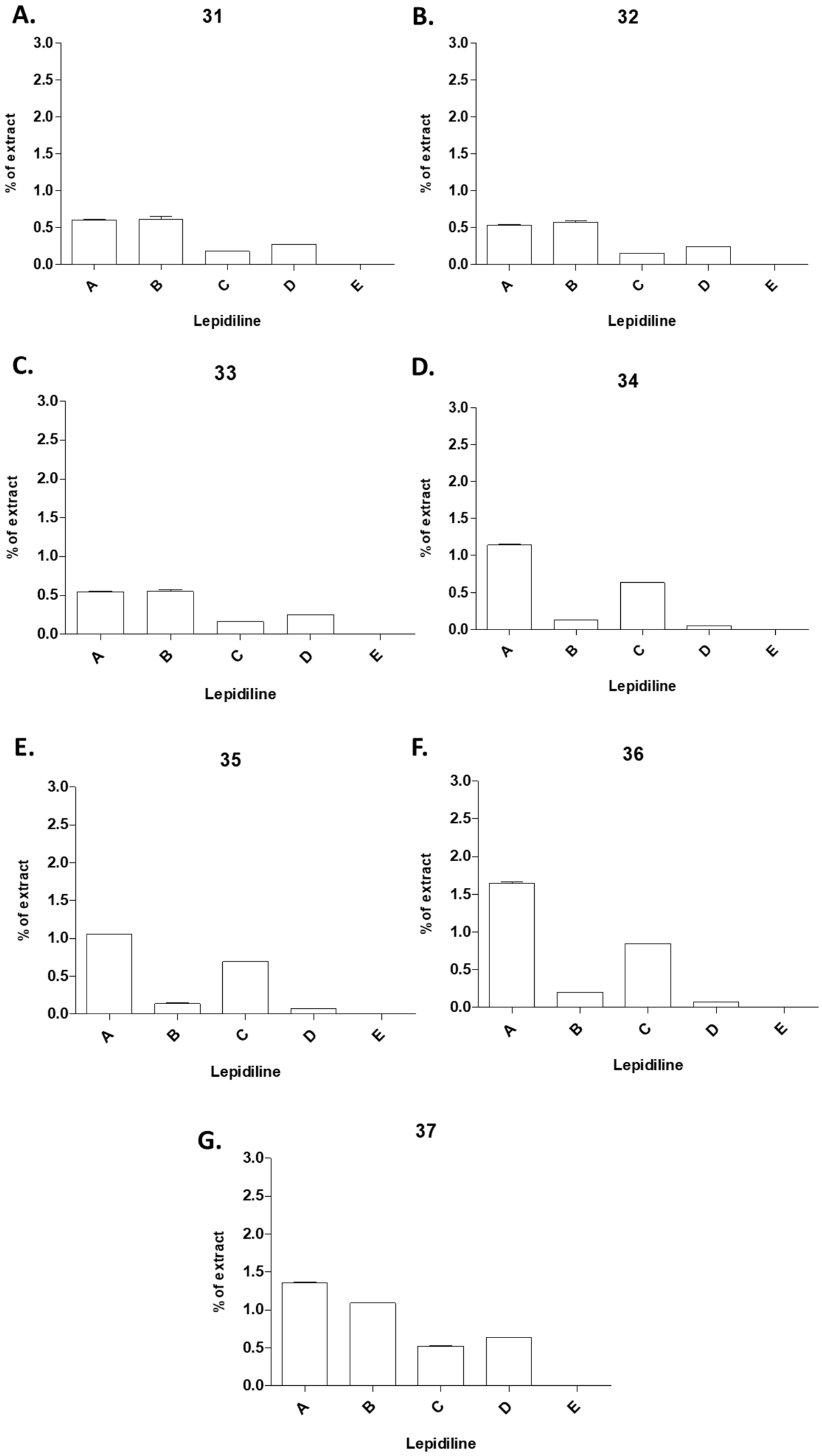

Figure S7. LC/MS (ESI+) MS/MS spectra of the Lepidiline A, B, C, and D.

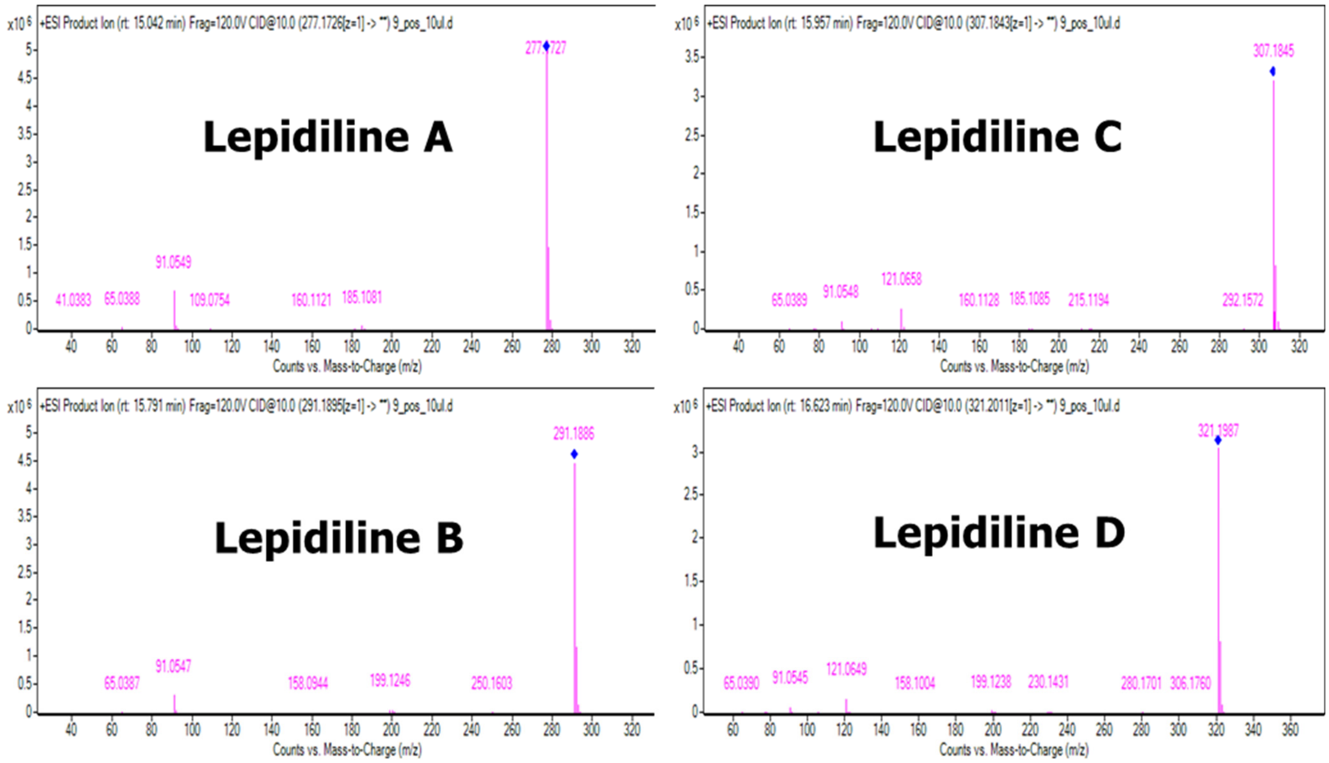

Supplement: Supplementary file 1 [file molecules-30-04360-s001.zip › molecules-3942351-supplementary.pdf]
